# Supplementary material for: Single Cell Transcriptomic and Chromatin Profiles Suggest Layer Vb Is the Only Layer With Shared Excitatory Cell Types in the Medial and Lateral Entorhinal Cortex
Source: Front Neural Circuits. 2022 Jan 26;15:806154. doi: 10.3389/fncir.2021.806154 (PMC8826650; doi:10.3389/fncir.2021.806154)
Supplement: Supplementary file 14 [file Data_Sheet_1.ZIP › Exc_vs_Inh_Motifs/knownResults.html]

Exc\_vs\_Inh\_Motifs/ - Homer Known Motif Enrichment Results


# Homer Known Motif Enrichment Results (Exc\_vs\_Inh\_Motifs/)

Homer *de novo* Motif Results  
Gene Ontology Enrichment Results  
Known Motif Enrichment Results (txt file)  
Total Target Sequences = 42183, Total Background Sequences = 10867

|  |  |  |  |  |  |  |  |  |  |  |  |
| --- | --- | --- | --- | --- | --- | --- | --- | --- | --- | --- | --- |
| Rank | Motif | Name | P-value | log P-pvalue | q-value (Benjamini) | # Target Sequences with Motif | % of Targets Sequences with Motif | # Background Sequences with Motif | % of Background Sequences with Motif | Motif File | SVG |
| 1 | A T G C T G C A A G T C C G T A A G T C A G T C A C G T A C T G A T G C G T C A | E2A(bHLH),near\_PU.1/Bcell-PU.1-ChIP-Seq(GSE21512)/Homer | 1e-3311 | -7.626e+03 | 0.0000 | 18999.0 | 45.04% | 2033.3 | 18.71% | motif file (matrix) | svg |
| 2 | T G C A G T A C C G T A A T C G A C T G A C G T C T A G C G A T T C G A A G T C | ZEB1(Zf)/PDAC-ZEB1-ChIP-Seq(GSE64557)/Homer | 1e-3075 | -7.082e+03 | 0.0000 | 20600.0 | 48.83% | 2433.9 | 22.39% | motif file (matrix) | svg |
| 3 | A G C T T C G A G T A C T C G A A T G C A T G C G C A T A T C G A G T C A G C T | Snail1(Zf)/LS174T-SNAIL1.HA-ChIP-Seq(GSE127183)/Homer | 1e-2675 | -6.162e+03 | 0.0000 | 13692.0 | 32.46% | 1295.5 | 11.92% | motif file (matrix) | svg |
| 4 | C T A G A C T G T G C A A G T C C G T A A C T G A C T G A C G T C T A G C G A T T A C G A G T C | ZEB2(Zf)/SNU398-ZEB2-ChIP-Seq(GSE103048)/Homer | 1e-2207 | -5.082e+03 | 0.0000 | 12596.0 | 29.86% | 1259.7 | 11.59% | motif file (matrix) | svg |
| 5 | T A C G C T A G T A C G A G T C C G T A A G T C A G T C A C G T A C T G A G T C G A T C T A G C | Slug(Zf)/Mesoderm-Snai2-ChIP-Seq(GSE61475)/Homer | 1e-2128 | -4.902e+03 | 0.0000 | 9753.0 | 23.12% | 825.5 | 7.59% | motif file (matrix) | svg |
| 6 | C T G A C T A G A T C G A G C T A C T G G A C T A G T C C T G A | Tbx5(T-box)/HL1-Tbx5.biotin-ChIP-Seq(GSE21529)/Homer | 1e-696 | -1.605e+03 | 0.0000 | 29681.0 | 70.36% | 6195.2 | 56.99% | motif file (matrix) | svg |
| 7 | C T A G C T G A T C A G A T C G A G C T C A T G G A C T A G T C C T G A T G C A | Tbx6(T-box)/ESC-Tbx6-ChIP-Seq(GSE93524)/Homer | 1e-632 | -1.456e+03 | 0.0000 | 13348.0 | 31.64% | 2224.0 | 20.46% | motif file (matrix) | svg |
| 8 | C T G A C T A G A C T G G C A T C T A G G C A T A T C G C T G A C G T A G T C A | Tbx21(T-box)/GM12878-TBX21-ChIP-Seq(Encode)/Homer | 1e-498 | -1.148e+03 | 0.0000 | 13671.0 | 32.41% | 2421.9 | 22.28% | motif file (matrix) | svg |
| 9 | T G A C A T G C C G T A A T C G A T G C C A G T C A T G A C T G A G T C G T A C | HEB(bHLH)/mES-Heb-ChIP-Seq(GSE53233)/Homer | 1e-488 | -1.124e+03 | 0.0000 | 23883.0 | 56.62% | 4904.8 | 45.12% | motif file (matrix) | svg |
| 10 | C T G A T G A C T G A C C G T A A C G T T G A C A G C T C T A G A C G T G A C T | Olig2(bHLH)/Neuron-Olig2-ChIP-Seq(GSE30882)/Homer | 1e-476 | -1.096e+03 | 0.0000 | 26258.0 | 62.25% | 5538.0 | 50.95% | motif file (matrix) | svg |
| 11 | C A G T T C A G G A T C A C T G A C G T C T A G A C T G A C T G G A C T C T A G | Egr1(Zf)/K562-Egr1-ChIP-Seq(GSE32465)/Homer | 1e-450 | -1.037e+03 | 0.0000 | 6569.0 | 15.57% | 947.8 | 8.72% | motif file (matrix) | svg |
| 12 | A C T G A G T C G T C A C G T A A G T C C G T A C T A G C T A G G A C T C A T G | SCRT1(Zf)/HEK293-SCRT1.eGFP-ChIP-Seq(Encode)/Homer | 1e-439 | -1.013e+03 | 0.0000 | 3453.0 | 8.19% | 381.0 | 3.50% | motif file (matrix) | svg |
| 13 | C G T A C T A G C A T G A G C T A C T G C G A T A T C G C G T A G T C A G T C A | Tbet(T-box)/CD8-Tbet-ChIP-Seq(GSE33802)/Homer | 1e-421 | -9.707e+02 | 0.0000 | 14239.0 | 33.75% | 2634.5 | 24.24% | motif file (matrix) | svg |
| 14 | T C G A A G T C C G T A A T C G T A G C A C G T A C T G A G C T A C G T A G T C | Ptf1a(bHLH)/Panc1-Ptf1a-ChIP-Seq(GSE47459)/Homer | 1e-402 | -9.264e+02 | 0.0000 | 29542.0 | 70.03% | 6519.6 | 59.98% | motif file (matrix) | svg |
| 15 | T C A G T G A C G T A C C G T A A C G T T G A C A C G T T C A G A G C T G A C T | NeuroD1(bHLH)/Islet-NeuroD1-ChIP-Seq(GSE30298)/Homer | 1e-385 | -8.876e+02 | 0.0000 | 14362.0 | 34.05% | 2705.1 | 24.89% | motif file (matrix) | svg |
| 16 | G C T A G A C T G A C T T G C A C G T A A G T C C G T A T A G C G A T C G A C T | Eomes(T-box)/H9-Eomes-ChIP-Seq(GSE26097)/Homer | 1e-373 | -8.589e+02 | 0.0000 | 24751.0 | 58.67% | 5286.5 | 48.63% | motif file (matrix) | svg |
| 17 | C G T A T G A C T C G A A G T C C G T A A T C G A T G C A C G T A C T G A G T C | E2A(bHLH)/proBcell-E2A-ChIP-Seq(GSE21978)/Homer | 1e-339 | -7.814e+02 | 0.0000 | 19528.0 | 46.29% | 4011.5 | 36.90% | motif file (matrix) | svg |
| 18 | C T G A C T G A C A T G A T C G A G C T A T C G G A C T C A T G C T G A G T C A | Tbr1(T-box)/Cortex-Tbr1-ChIP-Seq(GSE71384)/Homer | 1e-311 | -7.176e+02 | 0.0000 | 18295.0 | 43.37% | 3748.7 | 34.49% | motif file (matrix) | svg |
| 19 | G T A C G A T C C A G T A G T C A G T C A G T C T G C A G A T C C T G A A T G C G T C A A C G T | WT1(Zf)/Kidney-WT1-ChIP-Seq(GSE90016)/Homer | 1e-276 | -6.378e+02 | 0.0000 | 6198.0 | 14.69% | 1009.5 | 9.29% | motif file (matrix) | svg |
| 20 | T C G A T G A C G T A C C G T A C A G T T G A C A C G T A C T G A G C T A G C T | NeuroG2(bHLH)/Fibroblast-NeuroG2-ChIP-Seq(GSE75910)/Homer | 1e-276 | -6.359e+02 | 0.0000 | 22968.0 | 54.45% | 4979.3 | 45.81% | motif file (matrix) | svg |
| 21 | C T A G T C A G C A G T T C A G A C T G A C T G G A T C C T A G A C T G C T A G T C A G A T G C | KLF14(Zf)/HEK293-KLF14.GFP-ChIP-Seq(GSE58341)/Homer | 1e-247 | -5.688e+02 | 0.0000 | 11630.0 | 27.57% | 2250.9 | 20.71% | motif file (matrix) | svg |
| 22 | A T G C A G T C G T A C A G C T T C G A C T A G G A T C C T G A G T C A A G T C G C T A T C A G | Rfx5(HTH)/GM12878-Rfx5-ChIP-Seq(GSE31477)/Homer | 1e-225 | -5.196e+02 | 0.0000 | 4940.0 | 11.71% | 795.4 | 7.32% | motif file (matrix) | svg |
| 23 | G A C T C A G T A G C T C G A T A G T C G A T C A G T C C G T A A T G C T C A G | Rbpj1(?)/Panc1-Rbpj1-ChIP-Seq(GSE47459)/Homer | 1e-202 | -4.664e+02 | 0.0000 | 16761.0 | 39.73% | 3552.9 | 32.69% | motif file (matrix) | svg |
| 24 | A C G T T A C G G A T C A C T G A C G T C T A G A C T G A C T G G A T C C T A G C A T G C T A G | Egr2(Zf)/Thymocytes-Egr2-ChIP-Seq(GSE34254)/Homer | 1e-196 | -4.534e+02 | 0.0000 | 1563.0 | 3.71% | 171.9 | 1.58% | motif file (matrix) | svg |
| 25 | C T A G A G T C T A C G T A C G T G A C C G T A A C T G T A G C G C A T C A T G A T G C A G C T | Ascl1(bHLH)/NeuralTubes-Ascl1-ChIP-Seq(GSE55840)/Homer | 1e-181 | -4.182e+02 | 0.0000 | 20737.0 | 49.16% | 4587.7 | 42.21% | motif file (matrix) | svg |
| 26 | A G C T G C A T A C T G A C G T A G T C A C G T C T A G T A C G | Smad3(MAD)/NPC-Smad3-ChIP-Seq(GSE36673)/Homer | 1e-170 | -3.920e+02 | 0.0000 | 26404.0 | 62.59% | 6077.1 | 55.91% | motif file (matrix) | svg |
| 27 | G A C T C T A G A T G C A G T C G T C A T A C G A T G C A T C G | HIC1(Zf)/Treg-ZBTB29-ChIP-Seq(GSE99889)/Homer | 1e-168 | -3.871e+02 | 0.0000 | 19255.0 | 45.65% | 4241.2 | 39.02% | motif file (matrix) | svg |
| 28 | C T A G A G C T G A C T C A T G A G T C A G T C G T C A C A G T C T A G T C A G G T A C C T G A T C G A G A T C T G A C | Rfx2(HTH)/LoVo-RFX2-ChIP-Seq(GSE49402)/Homer | 1e-167 | -3.850e+02 | 0.0000 | 1955.0 | 4.63% | 254.6 | 2.34% | motif file (matrix) | svg |
| 29 | A G T C A T C G C T A G A G C T G A C T C T A G A G T C A G T C G C T A C A G T T C A G T C A G G A T C C T G A T C G A G A T C | RFX(HTH)/K562-RFX3-ChIP-Seq(SRA012198)/Homer | 1e-165 | -3.802e+02 | 0.0000 | 1742.0 | 4.13% | 217.4 | 2.00% | motif file (matrix) | svg |
| 30 | C G T A C T A G A C T G A C T G G A C T C T A G C A G T C T A G C A T G G A T C | KLF5(Zf)/LoVo-KLF5-ChIP-Seq(GSE49402)/Homer | 1e-157 | -3.632e+02 | 0.0000 | 7986.0 | 18.93% | 1542.9 | 14.19% | motif file (matrix) | svg |
| 31 | T A C G T A C G C T A G T C A G A G T C C G T A A T C G A T G C A C G T A C T G A G T C G A C T | Ascl2(bHLH)/ESC-Ascl2-ChIP-Seq(GSE97712)/Homer | 1e-151 | -3.480e+02 | 0.0000 | 15857.0 | 37.59% | 3431.9 | 31.57% | motif file (matrix) | svg |
| 32 | A G C T A C G T A C T G A T G C A G T C C G T A C T G A T A C G | NF1-halfsite(CTF)/LNCaP-NF1-ChIP-Seq(Unpublished)/Homer | 1e-148 | -3.420e+02 | 0.0000 | 19227.0 | 45.58% | 4277.4 | 39.35% | motif file (matrix) | svg |
| 33 | T A C G A T C G T A G C G A T C A C T G A C G T A G T C A C G T C T A G A T C G | Smad4(MAD)/ESC-SMAD4-ChIP-Seq(GSE29422)/Homer | 1e-148 | -3.417e+02 | 0.0000 | 16117.0 | 38.21% | 3501.8 | 32.22% | motif file (matrix) | svg |
| 34 | A C T G T C A G A G C T G A C T C A T G A G T C A G T C G C T A C G A T C T A G T C A G G T A C C T G A T C G A | Rfx1(HTH)/NPC-H3K4me1-ChIP-Seq(GSE16256)/Homer | 1e-143 | -3.314e+02 | 0.0000 | 3282.0 | 7.78% | 530.3 | 4.88% | motif file (matrix) | svg |
| 35 | A G C T C A T G G C A T G A T C T G C A C T A G G A T C A C G T | Tgif2(Homeobox)/mES-Tgif2-ChIP-Seq(GSE55404)/Homer | 1e-140 | -3.236e+02 | 0.0000 | 30953.0 | 73.38% | 7361.5 | 67.72% | motif file (matrix) | svg |
| 36 | G A C T C G A T T C A G G A T C G A C T A G C T A G C T A G T C G A T C C G T A C T A G C T A G T C G A T C G A C T G A | Bcl6(Zf)/Liver-Bcl6-ChIP-Seq(GSE31578)/Homer | 1e-140 | -3.226e+02 | 0.0000 | 16512.0 | 39.14% | 3617.5 | 33.28% | motif file (matrix) | svg |
| 37 | A T G C G A C T A C T G C A G T G A T C A C G T T A C G T A C G | Smad2(MAD)/ES-SMAD2-ChIP-Seq(GSE29422)/Homer | 1e-135 | -3.114e+02 | 0.0000 | 15680.0 | 37.17% | 3422.8 | 31.49% | motif file (matrix) | svg |
| 38 | C A G T T C A G A G C T G A C T A C G T A G T C G A T C G A C T C T G A A C T G G A T C C G T A C T G A A G T C G T A C | Rfx6(HTH)/Min6b1-Rfx6.HA-ChIP-Seq(GSE62844)/Homer | 1e-131 | -3.037e+02 | 0.0000 | 12168.0 | 28.85% | 2572.6 | 23.67% | motif file (matrix) | svg |
| 39 | A T G C G A T C C G A T A C G T A C G T A C T G C A G T A G C T | Sox3(HMG)/NPC-Sox3-ChIP-Seq(GSE33059)/Homer | 1e-131 | -3.029e+02 | 0.0000 | 18915.0 | 44.84% | 4238.3 | 38.99% | motif file (matrix) | svg |
| 40 | T C A G A C G T T C G A T A G C A G T C C G T A A C T G G T A C A C G T A C T G A T C G A G T C | Atoh1(bHLH)/Cerebellum-Atoh1-ChIP-Seq(GSE22111)/Homer | 1e-130 | -3.012e+02 | 0.0000 | 17522.0 | 41.54% | 3890.2 | 35.79% | motif file (matrix) | svg |
| 41 | A T G C G T A C C G T A A G C T G C A T T A C G A G C T A G C T A G T C A G C T | Sox6(HMG)/Myotubes-Sox6-ChIP-Seq(GSE32627)/Homer | 1e-129 | -2.988e+02 | 0.0000 | 18234.0 | 43.22% | 4071.2 | 37.45% | motif file (matrix) | svg |
| 42 | A T G C A G T C G C A T A G C T A C G T T C A G C G A T A G C T G A T C A T C G | Sox10(HMG)/SciaticNerve-Sox3-ChIP-Seq(GSE35132)/Homer | 1e-120 | -2.786e+02 | 0.0000 | 17882.0 | 42.39% | 4004.5 | 36.84% | motif file (matrix) | svg |
| 43 | T A G C G A T C G T A C C T G A G C A T C T G A C G T A T G C A C G T A G A T C | Hoxa13(Homeobox)/ChickenMSG-Hoxa13.Flag-ChIP-Seq(GSE86088)/Homer | 1e-116 | -2.693e+02 | 0.0000 | 28857.0 | 68.41% | 6854.7 | 63.06% | motif file (matrix) | svg |
| 44 | C A T G C T A G A G C T G A C T C A T G A G T C G A T C G C T A C G A T C T A G T C A G G T A C C T G A T C G A | X-box(HTH)/NPC-H3K4me1-ChIP-Seq(GSE16256)/Homer | 1e-114 | -2.634e+02 | 0.0000 | 2331.0 | 5.53% | 364.4 | 3.35% | motif file (matrix) | svg |
| 45 | T A G C G T A C C G T A C T A G A C T G T G C A C G T A A T G C C G T A A T C G | AR-halfsite(NR)/LNCaP-AR-ChIP-Seq(GSE27824)/Homer | 1e-113 | -2.613e+02 | 0.0000 | 31815.0 | 75.42% | 7661.0 | 70.48% | motif file (matrix) | svg |
| 46 | C T A G G T A C A G T C T G C A A G T C C T G A A G T C A G T C A G T C G C T A | Klf4(Zf)/mES-Klf4-ChIP-Seq(GSE11431)/Homer | 1e-109 | -2.525e+02 | 0.0000 | 2561.0 | 6.07% | 415.9 | 3.83% | motif file (matrix) | svg |
| 47 | T C A G G A C T C A G T C T G A A G C T C T A G G A C T T G C A C T G A A G T C | HLF(bZIP)/HSC-HLF.Flag-ChIP-Seq(GSE69817)/Homer | 1e-107 | -2.481e+02 | 0.0000 | 13481.0 | 31.96% | 2945.4 | 27.10% | motif file (matrix) | svg |
| 48 | A C G T A G T C A G T C C G A T A C G T A C G T A C T G A C G T A T G C G A C T A C T G T A C G | Sox21(HMG)/ESC-SOX21-ChIP-Seq(GSE110505)/Homer | 1e-101 | -2.340e+02 | 0.0000 | 19294.0 | 45.74% | 4412.0 | 40.59% | motif file (matrix) | svg |
| 49 | T A C G T G C A A G T C C G T A A C G T T G A C A C G T A C T G A C T G G C A T | TCF4(bHLH)/SHSY5Y-TCF4-ChIP-Seq(GSE96915)/Homer | 1e-96 | -2.214e+02 | 0.0000 | 22170.0 | 52.56% | 5161.2 | 47.48% | motif file (matrix) | svg |
| 50 | T A G C G C A T A G T C G A T C A T G C G A C T C T A G A C T G A C T G C T G A A C T G C T A G A G T C T G A C C G A T | GLIS3(Zf)/Thyroid-Glis3.GFP-ChIP-Seq(GSE103297)/Homer | 1e-95 | -2.207e+02 | 0.0000 | 10039.0 | 23.80% | 2138.6 | 19.67% | motif file (matrix) | svg |
| 51 | T C A G C T A G C T A G A C T G A C T G G A T C A C T G A C T G C T A G C T A G A G T C G A T C | KLF1(Zf)/HUDEP2-KLF1-CutnRun(GSE136251)/Homer | 1e-93 | -2.146e+02 | 0.0000 | 4991.0 | 11.83% | 963.1 | 8.86% | motif file (matrix) | svg |
| 52 | T C G A A C G T A C G T C T G A G A T C T C A G G A C T G T C A C G T A A G C T G T C A C T A G A G C T A C G T T C G A | NFIL3(bZIP)/HepG2-NFIL3-ChIP-Seq(Encode)/Homer | 1e-91 | -2.105e+02 | 0.0000 | 10781.0 | 25.56% | 2327.0 | 21.41% | motif file (matrix) | svg |
| 53 | G A C T G C A T C T A G C G A T G A T C T C G A C A T G G A T C | Tgif1(Homeobox)/mES-Tgif1-ChIP-Seq(GSE55404)/Homer | 1e-88 | -2.030e+02 | 0.0000 | 29696.0 | 70.40% | 7157.8 | 65.85% | motif file (matrix) | svg |
| 54 | C A G T C G A T G C A T G C A T G T C A A G C T C A T G C T A G A T G C G T A C | Hoxa11(Homeobox)/ChickenMSG-Hoxa11.Flag-ChIP-Seq(GSE86088)/Homer | 1e-86 | -1.991e+02 | 0.0000 | 27277.0 | 64.66% | 6520.7 | 59.99% | motif file (matrix) | svg |
| 55 | T C A G C A T G C A T G A C T G A C T G A G C T A C T G A C G T A C T G C A G T A T G C A G T C | KLF10(Zf)/HEK293-KLF10.GFP-ChIP-Seq(GSE58341)/Homer | 1e-85 | -1.974e+02 | 0.0000 | 6309.0 | 14.96% | 1278.1 | 11.76% | motif file (matrix) | svg |
| 56 | G T A C C A G T A C T G A C T G A C T G G A T C A C T G A C G T A C T G A C T G A G T C G A T C | KLF6(Zf)/PDAC-KLF6-ChIP-Seq(GSE64557)/Homer | 1e-85 | -1.967e+02 | 0.0000 | 6246.0 | 14.81% | 1264.2 | 11.63% | motif file (matrix) | svg |
| 57 | G A C T T C A G C T A G A G T C A G T C G T A C A G T C C T G A A G T C A G T C A G T C G A C T A G T C A C T G A T G C | KLF3(Zf)/MEF-Klf3-ChIP-Seq(GSE44748)/Homer | 1e-83 | -1.922e+02 | 0.0000 | 2751.0 | 6.52% | 483.2 | 4.45% | motif file (matrix) | svg |
| 58 | C T A G T A C G A G T C C G T A A G T C A C G T A G T C T C G A C G T A T A C G | Nkx2.1(Homeobox)/LungAC-Nkx2.1-ChIP-Seq(GSE43252)/Homer | 1e-82 | -1.909e+02 | 0.0000 | 25987.0 | 61.60% | 6192.7 | 56.97% | motif file (matrix) | svg |
| 59 | C T A G T A G C A G T C G T C A C T G A A C G T C G T A C G T A C G T A G C T A | Hoxd13(Homeobox)/ChickenMSG-Hoxd13.Flag-ChIP-Seq(GSE86088)/Homer | 1e-80 | -1.856e+02 | 0.0000 | 21445.0 | 50.84% | 5022.6 | 46.21% | motif file (matrix) | svg |
| 60 | C T G A T C G A C G T A A T G C C G T A C G T A C G A T C T A G T C A G G A T C | Sox15(HMG)/CPA-Sox15-ChIP-Seq(GSE62909)/Homer | 1e-80 | -1.849e+02 | 0.0000 | 12457.0 | 29.53% | 2764.1 | 25.43% | motif file (matrix) | svg |
| 61 | A G C T C T A G T G A C C G T A A C G T C G A T A G T C A G T C C T G A C A T G | TEAD3(TEA)/HepG2-TEAD3-ChIP-Seq(Encode)/Homer | 1e-76 | -1.770e+02 | 0.0000 | 13893.0 | 32.93% | 3128.2 | 28.78% | motif file (matrix) | svg |
| 62 | C T G A T C A G C A G T C T A G A C T G C T A G G A T C A T C G A C T G C T G A T C A G G A T C | Sp5(Zf)/mES-Sp5.Flag-ChIP-Seq(GSE72989)/Homer | 1e-75 | -1.745e+02 | 0.0000 | 6160.0 | 14.60% | 1263.6 | 11.62% | motif file (matrix) | svg |
| 63 | C T A G C T A G A G T C T C A G A C T G A C G T A C G T C T G A | MYB(HTH)/ERMYB-Myb-ChIPSeq(GSE22095)/Homer | 1e-75 | -1.743e+02 | 0.0000 | 18292.0 | 43.36% | 4234.4 | 38.96% | motif file (matrix) | svg |
| 64 | T C A G A G C T A C G T A C G T G T A C G A T C C G T A C T A G C A T G G T C A C G T A T C G A | STAT4(Stat)/CD4-Stat4-ChIP-Seq(GSE22104)/Homer | 1e-74 | -1.720e+02 | 0.0000 | 12828.0 | 30.41% | 2871.1 | 26.41% | motif file (matrix) | svg |
| 65 | C G A T C T A G A C G T A C G T A C G T C G T A A G C T C G A T A G C T C G T A C T A G T A G C | FoxD3(forkhead)/ZebrafishEmbryo-Foxd3.biotin-ChIP-seq(GSE106676)/Homer | 1e-71 | -1.644e+02 | 0.0000 | 12728.0 | 30.17% | 2856.5 | 26.28% | motif file (matrix) | svg |
| 66 | A G T C G A T C A G T C C G T A A T C G C A G T A G T C G T A C C T G A A C T G T C A G A G C T A G C T A G C T A G C T | PRDM15(Zf)/ESC-Prdm15-ChIP-Seq(GSE73694)/Homer | 1e-70 | -1.620e+02 | 0.0000 | 10739.0 | 25.46% | 2371.2 | 21.81% | motif file (matrix) | svg |
| 67 | T C A G A G C T G T A C C G T A A C G T C G T A C G T A C G T A G C T A G A C T | Cdx2(Homeobox)/mES-Cdx2-ChIP-Seq(GSE14586)/Homer | 1e-68 | -1.568e+02 | 0.0000 | 10155.0 | 24.07% | 2235.7 | 20.57% | motif file (matrix) | svg |
| 68 | A G T C A G T C C G A T A C G T A C G T A C T G A C G T A G C T A G T C A G T C | Sox4(HMG)/proB-Sox4-ChIP-Seq(GSE50066)/Homer | 1e-67 | -1.562e+02 | 0.0000 | 9633.0 | 22.84% | 2109.5 | 19.41% | motif file (matrix) | svg |
| 69 | T G A C C G A T A C T G A C T G A C T G G A C T A C T G A C G T A C T G A C T G G A T C G A T C | EKLF(Zf)/Erythrocyte-Klf1-ChIP-Seq(GSE20478)/Homer | 1e-66 | -1.527e+02 | 0.0000 | 1640.0 | 3.89% | 269.3 | 2.48% | motif file (matrix) | svg |
| 70 | T G A C G T A C C G T A A C T G T G A C C G A T A C T G A T C G A G C T T A C G T C G A T A G C G T A C C G T A A T C G T G A C G C A T A C T G A C T G A T G C | Twist(bHLH)/HMLE-TWIST1-ChIP-Seq(Chang\_et\_al)/Homer | 1e-66 | -1.525e+02 | 0.0000 | 2090.0 | 4.95% | 363.5 | 3.34% | motif file (matrix) | svg |
| 71 | G A C T C T A G C T A G A G T C T G C A A C T G A C G T A C G T C T A G T C A G | AMYB(HTH)/Testes-AMYB-ChIP-Seq(GSE44588)/Homer | 1e-66 | -1.523e+02 | 0.0000 | 16660.0 | 39.49% | 3853.9 | 35.45% | motif file (matrix) | svg |
| 72 | G C A T C T A G G T A C A G T C C G A T A C T G C T A G C T A G G T A C G C T A | ZNF416(Zf)/HEK293-ZNF416.GFP-ChIP-Seq(GSE58341)/Homer | 1e-65 | -1.506e+02 | 0.0000 | 12627.0 | 29.93% | 2849.3 | 26.21% | motif file (matrix) | svg |
| 73 | C T A G T C G A T G A C A G T C C G T A A C T G G T A C A C G T A C T G A C T G | BHLHA15(bHLH)/NIH3T3-BHLHB8.HA-ChIP-Seq(GSE119782)/Homer | 1e-64 | -1.485e+02 | 0.0000 | 20981.0 | 49.74% | 4957.1 | 45.60% | motif file (matrix) | svg |
| 74 | A T G C T C G A A G T C A G C T A C G T G T A C A G T C G C T A C T A G C A T G G T C A C T G A T C A G A G T C | Stat3+il21(Stat)/CD4-Stat3-ChIP-Seq(GSE19198)/Homer | 1e-63 | -1.452e+02 | 0.0000 | 8782.0 | 20.82% | 1916.7 | 17.63% | motif file (matrix) | svg |
| 75 | A G T C C T A G C T A G A G T C G A T C G T A C A G T C C T A G A G T C A G T C A G T C G T A C | Sp2(Zf)/HEK293-Sp2.eGFP-ChIP-Seq(Encode)/Homer | 1e-62 | -1.444e+02 | 0.0000 | 8492.0 | 20.13% | 1847.9 | 17.00% | motif file (matrix) | svg |
| 76 | A G T C A C G T A C T G A G C T A C G T A C G T G T C A A G T C | Foxo1(Forkhead)/RAW-Foxo1-ChIP-Seq(Fan\_et\_al.)/Homer | 1e-60 | -1.404e+02 | 0.0000 | 21637.0 | 51.29% | 5138.3 | 47.27% | motif file (matrix) | svg |
| 77 | G C T A T C G A C G T A C T A G A G C T G T C A G T C A C G T A A G T C C G T A | FOXA1(Forkhead)/LNCAP-FOXA1-ChIP-Seq(GSE27824)/Homer | 1e-60 | -1.401e+02 | 0.0000 | 16933.0 | 40.14% | 3940.1 | 36.25% | motif file (matrix) | svg |
| 78 | T C G A T C G A T A C G G A T C G T C A G T A C C G A T A G C T G T C A T G C A | Nkx3.1(Homeobox)/LNCaP-Nkx3.1-ChIP-Seq(GSE28264)/Homer | 1e-60 | -1.395e+02 | 0.0000 | 23511.0 | 55.73% | 5623.9 | 51.74% | motif file (matrix) | svg |
| 79 | T G A C C T A G T C A G G T C A C G T A T C A G C G A T T C A G T C G A T G C A C T G A T A G C | PU.1-IRF(ETS:IRF)/Bcell-PU.1-ChIP-Seq(GSE21512)/Homer | 1e-60 | -1.393e+02 | 0.0000 | 15693.0 | 37.20% | 3629.3 | 33.39% | motif file (matrix) | svg |
| 80 | A T G C G A T C C G T A A G C T C A G T A T C G G C A T A G C T G A C T A C T G | Sox17(HMG)/Endoderm-Sox17-ChIP-Seq(GSE61475)/Homer | 1e-60 | -1.382e+02 | 0.0000 | 8470.0 | 20.08% | 1849.4 | 17.01% | motif file (matrix) | svg |
| 81 | T G A C G C T A T C G A T G C A A G T C A G T C C G T A A G T C C G T A C T G A G C T A G T A C | RUNX2(Runt)/PCa-RUNX2-ChIP-Seq(GSE33889)/Homer | 1e-59 | -1.361e+02 | 0.0000 | 9545.0 | 22.63% | 2112.9 | 19.44% | motif file (matrix) | svg |
| 82 | A C G T C T A G A G C T A C G T A C G T C T G A A G T C G A C T A G C T C G T A | FOXM1(Forkhead)/MCF7-FOXM1-ChIP-Seq(GSE72977)/Homer | 1e-58 | -1.357e+02 | 0.0000 | 14534.0 | 34.45% | 3344.1 | 30.76% | motif file (matrix) | svg |
| 83 | C G A T C T G A C G T A C A G T A G T C G A T C G A T C A C T G | Pitx1(Homeobox)/Chicken-Pitx1-ChIP-Seq(GSE38910)/Homer | 1e-58 | -1.354e+02 | 0.0000 | 34572.0 | 81.96% | 8564.7 | 78.79% | motif file (matrix) | svg |
| 84 | A C T G T G A C G T A C C G T A A G T C T A C G A C G T A C T G G T C A A G T C | NPAS2(bHLH)/Liver-NPAS2-ChIP-Seq(GSE39860)/Homer | 1e-57 | -1.333e+02 | 0.0000 | 11620.0 | 27.55% | 2624.6 | 24.14% | motif file (matrix) | svg |
| 85 | C T G A A C G T A C G T A C G T A G T C G A C T C G A T C T G A A C T G C G T A C G T A T C G A | STAT5(Stat)/mCD4+-Stat5-ChIP-Seq(GSE12346)/Homer | 1e-55 | -1.274e+02 | 0.0000 | 5113.0 | 12.12% | 1062.9 | 9.78% | motif file (matrix) | svg |
| 86 | C G A T A C G T A C G T A C G T C G T A A G C T C A G T C T A G A T C G A C T G | HOXB13(Homeobox)/ProstateTumor-HOXB13-ChIP-Seq(GSE56288)/Homer | 1e-55 | -1.271e+02 | 0.0000 | 14646.0 | 34.72% | 3385.5 | 31.15% | motif file (matrix) | svg |
| 87 | T G C A A G C T A C G T C T A G G A T C C T A G G A T C G T C A C T G A A G T C | CEBP(bZIP)/ThioMac-CEBPb-ChIP-Seq(GSE21512)/Homer | 1e-54 | -1.255e+02 | 0.0000 | 8881.0 | 21.05% | 1964.6 | 18.07% | motif file (matrix) | svg |
| 88 | A G T C G A C T C A G T G T A C A G T C A T C G T C A G A C T G G T C A C G T A | Stat3(Stat)/mES-Stat3-ChIP-Seq(GSE11431)/Homer | 1e-54 | -1.248e+02 | 0.0000 | 5859.0 | 13.89% | 1241.0 | 11.42% | motif file (matrix) | svg |
| 89 | T G A C A G T C C G T A A C T G G T A C A C G T A C T G A C G T G A C T G A T C | Twist2(bHLH)/Myoblast-Twist2.Ty1-ChIP-Seq(GSE127998)/Homer | 1e-54 | -1.245e+02 | 0.0000 | 24160.0 | 57.27% | 5816.5 | 53.51% | motif file (matrix) | svg |
| 90 | A T G C C T G A G A C T A C G T A C G T G T A C G A T C C G A T C T A G C A T G C G T A C G T A C T G A G A C T | STAT1(Stat)/HelaS3-STAT1-ChIP-Seq(GSE12782)/Homer | 1e-53 | -1.226e+02 | 0.0000 | 4286.0 | 10.16% | 874.4 | 8.04% | motif file (matrix) | svg |
| 91 | A T G C A G T C G A T C C G T A A C G T A C G T A C T G A C G T A G C T G A T C | Sox2(HMG)/mES-Sox2-ChIP-Seq(GSE11431)/Homer | 1e-53 | -1.225e+02 | 0.0000 | 10013.0 | 23.74% | 2244.0 | 20.64% | motif file (matrix) | svg |
| 92 | T C A G T G A C G T A C T G C A G T A C C T A G G T A C A T G C A G T C G T C A A G T C G A C T | Klf9(Zf)/GBM-Klf9-ChIP-Seq(GSE62211)/Homer | 1e-52 | -1.206e+02 | 0.0000 | 2425.0 | 5.75% | 454.9 | 4.19% | motif file (matrix) | svg |
| 93 | G C T A T C G A C G T A C T A G A G C T G T C A G T C A C G T A A G T C C G T A | FOXA1(Forkhead)/MCF7-FOXA1-ChIP-Seq(GSE26831)/Homer | 1e-51 | -1.194e+02 | 0.0000 | 14410.0 | 34.16% | 3338.6 | 30.71% | motif file (matrix) | svg |
| 94 | G C T A C T G A T C G A A G T C A G T C C T G A A G T C G T C A C T G A T G C A | RUNX1(Runt)/Jurkat-RUNX1-ChIP-Seq(GSE29180)/Homer | 1e-51 | -1.181e+02 | 0.0000 | 11342.0 | 26.89% | 2577.4 | 23.71% | motif file (matrix) | svg |
| 95 | G A T C G C A T C T G A C G T A A G C T A G C T C G T A T C G A C T G A A C G T G C A T C G T A | Phox2a(Homeobox)/Neuron-Phox2a-ChIP-Seq(GSE31456)/Homer | 1e-50 | -1.166e+02 | 0.0000 | 6197.0 | 14.69% | 1329.6 | 12.23% | motif file (matrix) | svg |
| 96 | A T G C T C G A T A C G A C G T A T G C A G T C A C G T A G T C A G T C G A T C | Znf263(Zf)/K562-Znf263-ChIP-Seq(GSE31477)/Homer | 1e-50 | -1.161e+02 | 0.0000 | 14768.0 | 35.01% | 3433.3 | 31.58% | motif file (matrix) | svg |
| 97 | C G T A C G A T G A C T G A C T T G A C C T G A A T G C C T G A T A G C A G T C A C G T T C G A C A T G T A C G G A C T A T C G G A C T A C G T C T G A T C G A C G T A | Brachyury(T-box)/Mesoendoderm-Brachyury-ChIP-exo(GSE54963)/Homer | 1e-50 | -1.159e+02 | 0.0000 | 4117.0 | 9.76% | 841.9 | 7.74% | motif file (matrix) | svg |
| 98 | G C A T C G A T C T A G G C T A G C A T C G T A G A T C G C T A C G A T C G A T C T A G C G A T C G T A C G A T G A T C | DMRT1(DM)/Testis-DMRT1-ChIP-Seq(GSE64892)/Homer | 1e-49 | -1.133e+02 | 0.0000 | 3586.0 | 8.50% | 721.7 | 6.64% | motif file (matrix) | svg |
| 99 | T G A C A G T C C T G A T G A C C G T A A C G T A C G T A G T C A G T C C G T A | TEAD1(TEAD)/HepG2-TEAD1-ChIP-Seq(Encode)/Homer | 1e-49 | -1.129e+02 | 0.0000 | 11663.0 | 27.65% | 2664.7 | 24.51% | motif file (matrix) | svg |
| 100 | A C G T C T A G C G T A A G T C G T A C A C G T A C G T A C G T G T C A G T A C T G A C G A C T | Nur77(NR)/K562-NR4A1-ChIP-Seq(GSE31363)/Homer | 1e-49 | -1.129e+02 | 0.0000 | 2402.0 | 5.69% | 454.5 | 4.18% | motif file (matrix) | svg |
| 101 | G A C T C G A T C A T G G C T A G C A T C G T A A G T C G C T A G C A T C G A T T A C G G C A T C G T A C A G T G A T C | DMRT6(DM)/Testis-DMRT6-ChIP-Seq(GSE60440)/Homer | 1e-48 | -1.108e+02 | 0.0000 | 2865.0 | 6.79% | 559.1 | 5.14% | motif file (matrix) | svg |
| 102 | A G T C A T C G G C A T C A T G A C T G A T C G C G A T C T A G A C T G A G C T T G A C G A C T | Gli2(Zf)/GM2-Gli2-ChIP-Chip(GSE112702)/Homer | 1e-47 | -1.087e+02 | 0.0000 | 1994.0 | 4.73% | 367.6 | 3.38% | motif file (matrix) | svg |
| 103 | A G T C G T A C A G C T C T A G A G T C C G A T A C T G C G T A A C T G G T C A | Zic(Zf)/Cerebellum-ZIC1.2-ChIP-Seq(GSE60731)/Homer | 1e-46 | -1.070e+02 | 0.0000 | 8431.0 | 19.99% | 1879.2 | 17.29% | motif file (matrix) | svg |
| 104 | A T G C G A T C G A C T A G C T C G A T C G A T G T C A C G A T T C G A A T C G T A G C T A G C | TATA-Box(TBP)/Promoter/Homer | 1e-46 | -1.068e+02 | 0.0000 | 17066.0 | 40.46% | 4028.1 | 37.06% | motif file (matrix) | svg |
| 105 | T C A G T A C G T A G C A C G T A C T G C G A T A G T C C G T A T A C G A G T C | Meis1(Homeobox)/MastCells-Meis1-ChIP-Seq(GSE48085)/Homer | 1e-46 | -1.064e+02 | 0.0000 | 17745.0 | 42.07% | 4201.3 | 38.65% | motif file (matrix) | svg |
| 106 | C A G T A C T G T C A G T G C A G C T A A T G C T C G A A T C G G T C A T G C A | ZNF189(Zf)/HEK293-ZNF189.GFP-ChIP-Seq(GSE58341)/Homer | 1e-43 | -1.008e+02 | 0.0000 | 9435.0 | 22.37% | 2133.8 | 19.63% | motif file (matrix) | svg |
| 107 | C T A G C A G T T G A C C G T A G A T C T C A G G A C T C A T G | BMAL1(bHLH)/Liver-Bmal1-ChIP-Seq(GSE39860)/Homer | 1e-43 | -1.002e+02 | 0.0000 | 18683.0 | 44.29% | 4451.7 | 40.95% | motif file (matrix) | svg |
| 108 | T C A G T A C G G A T C G T A C T C G A C G A T C T G A C G T A G C T A C G T A | Hoxd11(Homeobox)/ChickenMSG-Hoxd11.Flag-ChIP-Seq(GSE86088)/Homer | 1e-42 | -9.862e+01 | 0.0000 | 27165.0 | 64.40% | 6646.9 | 61.15% | motif file (matrix) | svg |
| 109 | T A G C T A G C G C A T C A T G A C T G G C T A C G T A A C G T A C T G G A T C | TEAD4(TEA)/Tropoblast-Tead4-ChIP-Seq(GSE37350)/Homer | 1e-42 | -9.808e+01 | 0.0000 | 9691.0 | 22.97% | 2200.8 | 20.25% | motif file (matrix) | svg |
| 110 | A T C G G A T C G C A T C G T A G T C A A C G T A T G C A G T C | CRX(Homeobox)/Retina-Crx-ChIP-Seq(GSE20012)/Homer | 1e-42 | -9.740e+01 | 0.0000 | 24103.0 | 57.14% | 5850.3 | 53.82% | motif file (matrix) | svg |
| 111 | C A T G A C T G C T A G T C G A T C G A T C G A T C G A T C A G T C A G T C A G T G A C T G A C C G T A A C T G T G C A C G A T A C T G | RBPJ:Ebox(?,bHLH)/Panc1-Rbpj1-ChIP-Seq(GSE47459)/Homer | 1e-41 | -9.560e+01 | 0.0000 | 4384.0 | 10.39% | 923.4 | 8.49% | motif file (matrix) | svg |
| 112 | A G T C G C A T C G T A C G T A G T A C A C G T A C T G G A T C G A T C T C G A | BMYB(HTH)/Hela-BMYB-ChIP-Seq(GSE27030)/Homer | 1e-41 | -9.477e+01 | 0.0000 | 16877.0 | 40.01% | 4002.8 | 36.82% | motif file (matrix) | svg |
| 113 | A T C G G A C T C G A T C G T A C G T A C A G T G A T C G A T C G A T C A G C T | Otx2(Homeobox)/EpiLC-Otx2-ChIP-Seq(GSE56098)/Homer | 1e-40 | -9.412e+01 | 0.0000 | 9581.0 | 22.71% | 2179.6 | 20.05% | motif file (matrix) | svg |
| 114 | G C A T G C A T C T G A A C G T C T G A A C G T C G T A C G T A C G T A A G T C G T C A G T C A | Foxf1(Forkhead)/Lung-Foxf1-ChIP-Seq(GSE77951)/Homer | 1e-40 | -9.342e+01 | 0.0000 | 14220.0 | 33.71% | 3334.5 | 30.68% | motif file (matrix) | svg |
| 115 | T A C G T C A G G A T C G T A C T C G A G A C T G C T A G C T A G C T A C G T A G A T C G T C A | CDX4(Homeobox)/ZebrafishEmbryos-Cdx4.Myc-ChIP-Seq(GSE48254)/Homer | 1e-39 | -9.046e+01 | 0.0000 | 12790.0 | 30.32% | 2981.9 | 27.43% | motif file (matrix) | svg |
| 116 | T G A C C T G A C T A G T C G A C T G A A T G C C G T A A C T G G C A T G T A C G C A T A T C G G C A T A G C T G A T C | PR(NR)/T47D-PR-ChIP-Seq(GSE31130)/Homer | 1e-39 | -9.035e+01 | 0.0000 | 23266.0 | 55.15% | 5647.4 | 51.95% | motif file (matrix) | svg |
| 117 | A T G C G A C T A C G T C T A G A C G T A C G T A C G T C T G A G A T C G C T A A G C T C G T A | Foxa2(Forkhead)/Liver-Foxa2-ChIP-Seq(GSE25694)/Homer | 1e-39 | -9.033e+01 | 0.0000 | 11171.0 | 26.48% | 2578.3 | 23.72% | motif file (matrix) | svg |
| 118 | C G A T T G C A G T A C C G T A A G T C C T A G G A C T C A T G | NPAS(bHLH)/Liver-NPAS-ChIP-Seq(GSE39860)/Homer | 1e-39 | -9.000e+01 | 0.0000 | 16869.0 | 39.99% | 4009.8 | 36.89% | motif file (matrix) | svg |
| 119 | C T G A T C A G G T A C T G C A A G T C C G T A A G T C A C T G A C G T A C T G | MNT(bHLH)/HepG2-MNT-ChIP-Seq(Encode)/Homer | 1e-38 | -8.973e+01 | 0.0000 | 11682.0 | 27.69% | 2706.9 | 24.90% | motif file (matrix) | svg |
| 120 | C G A T C T A G T C A G C A G T C G T A A G T C G C T A A C G T G A C T A T G C A G T C G C T A | PRDM10(Zf)/HEK293-PRDM10.eGFP-ChIP-Seq(Encode)/Homer | 1e-38 | -8.892e+01 | 0.0000 | 6050.0 | 14.34% | 1327.6 | 12.21% | motif file (matrix) | svg |
| 121 | A G C T T G A C C G A T C G A T C T A G A C G T C A G T C A G T G C T A A G T C | FOXK1(Forkhead)/HEK293-FOXK1-ChIP-Seq(GSE51673)/Homer | 1e-38 | -8.804e+01 | 0.0000 | 14352.0 | 34.02% | 3377.8 | 31.07% | motif file (matrix) | svg |
| 122 | T C A G C T A G C T A G T G C A C G A T C G A T C G T A C T A G | GSC(Homeobox)/FrogEmbryos-GSC-ChIP-Seq(DRA000576)/Homer | 1e-36 | -8.491e+01 | 0.0000 | 14114.0 | 33.46% | 3323.4 | 30.57% | motif file (matrix) | svg |
| 123 | C T G A C T A G A T C G G C A T A C T G G T A C A T G C C G T A A C T G G C T A A G T C C G T A | Tbox:Smad(T-box,MAD)/ESCd5-Smad2\_3-ChIP-Seq(GSE29422)/Homer | 1e-36 | -8.306e+01 | 0.0000 | 2229.0 | 5.28% | 437.8 | 4.03% | motif file (matrix) | svg |
| 124 | C T G A A G C T A C G T A C G T A G T C G A C T G A C T C T G A C T G A C T A G C G T A C G T A | STAT6(Stat)/CD4-Stat6-ChIP-Seq(GSE22104)/Homer | 1e-35 | -8.085e+01 | 0.0000 | 8071.0 | 19.13% | 1829.0 | 16.83% | motif file (matrix) | svg |
| 125 | G T A C G C A T C T G A C G T A G A C T A G C T C A T G T G C A C T G A A C G T G A C T C G T A | Prop1(Homeobox)/GHFT1-PROP1.biotin-ChIP-Seq(GSE77302)/Homer | 1e-34 | -8.055e+01 | 0.0000 | 9768.0 | 23.16% | 2247.8 | 20.68% | motif file (matrix) | svg |
| 126 | T C G A T G A C G C A T A G C T C A G T G A T C G C T A G A T C G A C T A C G T G C A T A G T C | PRDM1(Zf)/Hela-PRDM1-ChIP-Seq(GSE31477)/Homer | 1e-34 | -7.900e+01 | 0.0000 | 7138.0 | 16.92% | 1604.8 | 14.76% | motif file (matrix) | svg |
| 127 | T A C G A T C G G A T C G T C A C T G A G C A T C G A T G C T A T C G A G C T A | Unknown(Homeobox)/Limb-p300-ChIP-Seq/Homer | 1e-33 | -7.633e+01 | 0.0000 | 9670.0 | 22.92% | 2230.6 | 20.52% | motif file (matrix) | svg |
| 128 | C T G A C A G T C T G A A G T C C T A G G A C T A T C G G T A C | HIF-1b(HLH)/T47D-HIF1b-ChIP-Seq(GSE59937)/Homer | 1e-32 | -7.584e+01 | 0.0000 | 9351.0 | 22.17% | 2152.3 | 19.80% | motif file (matrix) | svg |
| 129 | C T G A C T G A T A G C G A T C G C T A G T A C A C G T G A T C T G C A C G T A | Nkx2.5(Homeobox)/HL1-Nkx2.5.biotin-ChIP-Seq(GSE21529)/Homer | 1e-32 | -7.564e+01 | 0.0000 | 21527.0 | 51.03% | 5229.0 | 48.11% | motif file (matrix) | svg |
| 130 | A T G C C T G A A T C G T A C G A G T C C G A T T C A G C G A T C T A G A G C T G T C A G T C A C G T A A G T C C G T A T A C G C T G A | Fox:Ebox(Forkhead,bHLH)/Panc1-Foxa2-ChIP-Seq(GSE47459)/Homer | 1e-32 | -7.556e+01 | 0.0000 | 13533.0 | 32.08% | 3195.0 | 29.39% | motif file (matrix) | svg |
| 131 | T C G A T G A C A G T C C G T A A G T C C T A G A C G T A C T G A C T G A G C T A G T C G C A T | Max(bHLH)/K562-Max-ChIP-Seq(GSE31477)/Homer | 1e-32 | -7.500e+01 | 0.0000 | 7228.0 | 17.13% | 1632.4 | 15.02% | motif file (matrix) | svg |
| 132 | A G C T G A C T C T A G C G T A C A T G C G A T C T A G A T C G G A C T C A G T | Bapx1(Homeobox)/VertebralCol-Bapx1-ChIP-Seq(GSE36672)/Homer | 1e-32 | -7.469e+01 | 0.0000 | 21138.0 | 50.11% | 5131.2 | 47.21% | motif file (matrix) | svg |
| 133 | C G A T C T A G C T G A A T G C C T G A T C G A C G T A C T G A T C G A T A G C A G T C C G T A A C T G T C G A A T G C | Hand2(bHLH)/Mesoderm-Hand2-ChIP-Seq(GSE61475)/Homer | 1e-32 | -7.410e+01 | 0.0000 | 5756.0 | 13.64% | 1277.0 | 11.75% | motif file (matrix) | svg |
| 134 | A G T C G A C T A C T G G A T C G T A C C G T A T G A C A G T C C G A T A G C T A C G T A C G T C T A G G A C T C T G A | ZNF7(Zf)/HepG2-ZNF7.Flag-ChIP-Seq(Encode)/Homer | 1e-31 | -7.360e+01 | 0.0000 | 7526.0 | 17.84% | 1707.6 | 15.71% | motif file (matrix) | svg |
| 135 | C G T A G C T A C G A T C T A G A C G T G T C A C G T A C G T A A G T C C G T A T G C A T A C G | FoxL2(Forkhead)/Ovary-FoxL2-ChIP-Seq(GSE60858)/Homer | 1e-31 | -7.255e+01 | 0.0000 | 12884.0 | 30.54% | 3038.9 | 27.96% | motif file (matrix) | svg |
| 136 | C G T A A C G T A C T G G T A C C G T A A C G T C G T A C G T A A C G T A C T G A G T C C G T A A C G T C T G A G C A T | OCT:OCT-short(POU,Homeobox)/NPC-OCT6-ChIP-Seq(GSE43916)/Homer | 1e-31 | -7.228e+01 | 0.0000 | 11323.0 | 26.84% | 2648.7 | 24.37% | motif file (matrix) | svg |
| 137 | G A C T C A G T G A T C G A T C A C G T G A T C C T G A T A C G C G T A G T C A | STAT6(Stat)/Macrophage-Stat6-ChIP-Seq(GSE38377)/Homer | 1e-30 | -6.997e+01 | 0.0000 | 8150.0 | 19.32% | 1866.5 | 17.17% | motif file (matrix) | svg |
| 138 | C T A G C G T A G T C A C G T A A G T C G A T C A G C T C T A G C G T A A C G T G T C A G A T C | Six2(Homeobox)/NephronProgenitor-Six2-ChIP-Seq(GSE39837)/Homer | 1e-29 | -6.781e+01 | 0.0000 | 12176.0 | 28.86% | 2870.6 | 26.41% | motif file (matrix) | svg |
| 139 | T G C A G C A T A G C T G C A T A G T C A G T C A G T C C T G A A C T G T C G A T C G A C A G T A T C G A G T C G A T C | ZNF143\|STAF(Zf)/CUTLL-ZNF143-ChIP-Seq(GSE29600)/Homer | 1e-29 | -6.764e+01 | 0.0000 | 3498.0 | 8.29% | 745.3 | 6.86% | motif file (matrix) | svg |
| 140 | C G T A C A T G C A T G A C T G C T A G T C G A G C A T C G A T A G C T A G T C G A T C G T A C | NFkB-p65(RHD)/GM12787-p65-ChIP-Seq(GSE19485)/Homer | 1e-29 | -6.700e+01 | 0.0000 | 4814.0 | 11.41% | 1059.5 | 9.75% | motif file (matrix) | svg |
| 141 | G T C A T C G A C T A G C T A G A G T C G T C A C G A T C T A G G A C T G A T C G A T C T C A G C T A G C T G A A G T C G C T A C A G T T C A G G A T C G A T C | p63(p53)/Keratinocyte-p63-ChIP-Seq(GSE17611)/Homer | 1e-29 | -6.680e+01 | 0.0000 | 3566.0 | 8.45% | 762.6 | 7.02% | motif file (matrix) | svg |
| 142 | C T G A T C A G G T A C G C T A A C T G T G A C G C A T C A T G | SCL(bHLH)/HPC7-Scl-ChIP-Seq(GSE13511)/Homer | 1e-28 | -6.632e+01 | 0.0000 | 35447.0 | 84.03% | 8909.9 | 81.97% | motif file (matrix) | svg |
| 143 | A T G C G A T C C G A T C T A G A C T G G C T A C G T A A G C T A C T G A G C T | TEAD2(TEA)/Py2T-Tead2-ChIP-Seq(GSE55709)/Homer | 1e-27 | -6.349e+01 | 0.0000 | 6252.0 | 14.82% | 1412.8 | 13.00% | motif file (matrix) | svg |
| 144 | C T A G A C T G C T A G T C A G T C A G T A C G C T A G A C T G | Maz(Zf)/HepG2-Maz-ChIP-Seq(GSE31477)/Homer | 1e-27 | -6.344e+01 | 0.0000 | 8035.0 | 19.05% | 1849.1 | 17.01% | motif file (matrix) | svg |
| 145 | A G C T A T G C G A C T G C A T C G T A A G C T G T A C C G A T A T C G A G T C | Gata6(Zf)/HUG1N-GATA6-ChIP-Seq(GSE51936)/Homer | 1e-27 | -6.342e+01 | 0.0000 | 10949.0 | 25.96% | 2572.2 | 23.66% | motif file (matrix) | svg |
| 146 | C T A G C T A G G C A T C G A T C T G A G T C A G C T A A T G C C G T A C A G T G A C T C G T A T G C A | Hnf1(Homeobox)/Liver-Foxa2-Chip-Seq(GSE25694)/Homer | 1e-27 | -6.252e+01 | 0.0000 | 2000.0 | 4.74% | 402.0 | 3.70% | motif file (matrix) | svg |
| 147 | G A T C G T A C C G A T A C T G A C T G C G T A C G T A A C G T A C T G G A T C | TEAD(TEA)/Fibroblast-PU.1-ChIP-Seq(Unpublished)/Homer | 1e-26 | -6.211e+01 | 0.0000 | 8540.0 | 20.24% | 1976.9 | 18.19% | motif file (matrix) | svg |
| 148 | C G A T A C G T C G T A C G T A A C G T A G C T T G C A C T G A C G T A A C G T A C G T C G T A | Phox2b(Homeobox)/CLBGA-PHOX2B-ChIP-Seq(GSE90683)/Homer | 1e-26 | -6.092e+01 | 0.0000 | 3249.0 | 7.70% | 694.6 | 6.39% | motif file (matrix) | svg |
| 149 | G C T A A C G T C T A G G T A C G C T A G A C T C T G A G C A T C A T G G A T C | Pit1(Homeobox)/GCrat-Pit1-ChIP-Seq(GSE58009)/Homer | 1e-26 | -6.051e+01 | 0.0000 | 15769.0 | 37.38% | 3792.3 | 34.89% | motif file (matrix) | svg |
| 150 | C G A T C T A G A C G T G T C A C G T A C G T A A G T C C G T A | Foxo3(Forkhead)/U2OS-Foxo3-ChIP-Seq(E-MTAB-2701)/Homer | 1e-25 | -5.832e+01 | 0.0000 | 10883.0 | 25.80% | 2566.7 | 23.61% | motif file (matrix) | svg |
| 151 | C G T A C T G A C T A G C G T A C G T A A G T C C G T A C A G T G C A T G T C A C G A T A C T G A C G T G C A T G A T C | PGR(NR)/EndoStromal-PGR-ChIP-Seq(GSE69539)/Homer | 1e-25 | -5.763e+01 | 0.0000 | 3759.0 | 8.91% | 820.0 | 7.54% | motif file (matrix) | svg |
| 152 | A G C T A G T C A T G C A G C T A C G T C G T A A C G T A G T C C G A T A T G C | Gata2(Zf)/K562-GATA2-ChIP-Seq(GSE18829)/Homer | 1e-24 | -5.754e+01 | 0.0000 | 7651.0 | 18.14% | 1765.9 | 16.25% | motif file (matrix) | svg |
| 153 | C A G T G A C T A G T C G C T A C T G A G T C A C G T A A G C T G T C A C T G A C T G A C T G A A C T G A G C T G A T C | MYNN(Zf)/HEK293-MYNN.eGFP-ChIP-Seq(Encode)/Homer | 1e-24 | -5.682e+01 | 0.0000 | 3851.0 | 9.13% | 842.9 | 7.75% | motif file (matrix) | svg |
| 154 | T A G C G C T A T C G A C T G A A G T C A G T C C T G A A G T C C G T A C T A G | RUNX(Runt)/HPC7-Runx1-ChIP-Seq(GSE22178)/Homer | 1e-23 | -5.513e+01 | 0.0000 | 7564.0 | 17.93% | 1748.2 | 16.08% | motif file (matrix) | svg |
| 155 | C T G A C T G A C T G A A T G C G A T C C A T G A C T G G A C T G A C T G C A T C G T A C G T A A G T C G T A C C T G A A T C G G C A T G A C T G A C T A G C T | GRHL2(CP2)/HBE-GRHL2-ChIP-Seq(GSE46194)/Homer | 1e-23 | -5.408e+01 | 0.0000 | 4402.0 | 10.44% | 978.7 | 9.00% | motif file (matrix) | svg |
| 156 | T C G A C G T A A G T C A G C T C G T A A G T C T C G A G C T A G A C T C G A T A G T C A G T C A G T C C T G A T C A G T G C A T C G A C A G T A T C G A G T C | GFY-Staf(?,Zf)/Promoter/Homer | 1e-21 | -5.058e+01 | 0.0000 | 334.0 | 0.79% | 48.3 | 0.44% | motif file (matrix) | svg |
| 157 | A G T C T A G C G A C T A C G T C T A G A C G T A C G T A C G T C T G A A G T C G C T A G A C T C G T A C T A G A C T G | Foxa3(Forkhead)/Liver-Foxa3-ChIP-Seq(GSE77670)/Homer | 1e-21 | -4.993e+01 | 0.0000 | 4872.0 | 11.55% | 1098.9 | 10.11% | motif file (matrix) | svg |
| 158 | C G T A C T G A T A C G G A C T G A T C T C G A G A T C G A T C T G C A G A C T T C G A C G T A A T C G A G T C C G A T C G T A C G T A G T C A C G T A C T A G | PSE(SNAPc)/K562-mStart-Seq/Homer | 1e-21 | -4.983e+01 | 0.0000 | 7053.0 | 16.72% | 1632.8 | 15.02% | motif file (matrix) | svg |
| 159 | T G A C T A G C T C A G T C G A T C G A C G T A A G T C C G T A C G T A C G A T C T A G T A C G | Sox7(HMG)/ESC-Sox7-ChIP-Seq(GSE133899)/Homer | 1e-20 | -4.777e+01 | 0.0000 | 3256.0 | 7.72% | 712.7 | 6.56% | motif file (matrix) | svg |
| 160 | A G T C G A C T C A G T A C T G C T A G T G A C G C T A A T G C G C A T A T C G C G A T A C T G G A T C G T A C G T C A C T G A | NF1(CTF)/LNCAP-NF1-ChIP-Seq(Unpublished)/Homer | 1e-20 | -4.775e+01 | 0.0000 | 4313.0 | 10.22% | 966.9 | 8.90% | motif file (matrix) | svg |
| 161 | C T A G C T G A A G T C G C T A C G A T A C T G G A C T G A T C G A T C C T G A C T A G C T G A T G A C G C T A C G A T T C A G G A C T G A T C G A T C T G A C | p53(p53)/Saos-p53-ChIP-Seq(GSE15780)/Homer | 1e-20 | -4.651e+01 | 0.0000 | 1038.0 | 2.46% | 198.8 | 1.83% | motif file (matrix) | svg |
| 162 | C T A G C T G A A G T C G C T A C G A T A C T G G A C T G A T C G A T C C T G A C T A G C T G A T G A C G C T A C G A T T C A G G A C T G A T C G A T C T G A C | p53(p53)/Saos-p53-ChIP-Seq/Homer | 1e-20 | -4.651e+01 | 0.0000 | 1038.0 | 2.46% | 198.8 | 1.83% | motif file (matrix) | svg |
| 163 | T C A G T C A G T A G C A G T C C T G A A G T C C T A G A C G T A C T G A T C G | c-Myc(bHLH)/mES-cMyc-ChIP-Seq(GSE11431)/Homer | 1e-20 | -4.637e+01 | 0.0000 | 4807.0 | 11.40% | 1088.6 | 10.01% | motif file (matrix) | svg |
| 164 | C G T A A C G T A C G T A C G T A C G T A G T C A G T C C T G A A G C T A G C T | NFAT(RHD)/Jurkat-NFATC1-ChIP-Seq(Jolma\_et\_al.)/Homer | 1e-20 | -4.606e+01 | 0.0000 | 12059.0 | 28.59% | 2889.8 | 26.59% | motif file (matrix) | svg |
| 165 | T A G C G C T A A C T G C G T A A C G T C G T A C G T A T A C G T C A G T C G A | Gata1(Zf)/K562-GATA1-ChIP-Seq(GSE18829)/Homer | 1e-19 | -4.444e+01 | 0.0000 | 6605.0 | 15.66% | 1532.5 | 14.10% | motif file (matrix) | svg |
| 166 | C T G A T C A G G C T A A G C T A G T C G A C T C T G A C T A G T G C A C T G A A G T C G T A C G A T C A C T G T C G A | ZBTB12(Zf)/HEK293-ZBTB12.GFP-ChIP-Seq(GSE58341)/Homer | 1e-19 | -4.423e+01 | 0.0000 | 4191.0 | 9.94% | 942.8 | 8.67% | motif file (matrix) | svg |
| 167 | G A T C G C A T A C T G C T A G C T G A A G C T G C T A G C T A C T G A T C A G G C A T T G C A A C G T A C G T G A T C G A C T G C A T C T A G T A C G G A C T C T A G C A T G C T A G G T A C T C G A | ZNF136(Zf)/HEK293-ZNF136.GFP-ChIP-Seq(GSE58341)/Homer | 1e-18 | -4.355e+01 | 0.0000 | 1497.0 | 3.55% | 304.9 | 2.80% | motif file (matrix) | svg |
| 168 | C G T A C T A G G A C T G T C A G T C A C G T A A G T C C G T A T C G A T C G A T C G A C G T A C T G A C T A G G C T A C G T A T A G C C G T A C G A T C G T A | FOXA1:AR(Forkhead,NR)/LNCAP-AR-ChIP-Seq(GSE27824)/Homer | 1e-18 | -4.186e+01 | 0.0000 | 1143.0 | 2.71% | 225.1 | 2.07% | motif file (matrix) | svg |
| 169 | C T A G A C T G T G C A G T C A A T G C C G T A A T C G A T G C A G T C C T A G | ZNF341(Zf)/EBV-ZNF341-ChIP-Seq(GSE113194)/Homer | 1e-17 | -4.114e+01 | 0.0000 | 7511.0 | 17.81% | 1763.6 | 16.22% | motif file (matrix) | svg |
| 170 | A T G C G A C T A G C T C T A G C G T A C T A G C G A T C T A G A T C G G A T C | Nkx2.2(Homeobox)/NPC-Nkx2.2-ChIP-Seq(GSE61673)/Homer | 1e-17 | -4.062e+01 | 0.0000 | 18526.0 | 43.92% | 4548.0 | 41.84% | motif file (matrix) | svg |
| 171 | G C A T C T G A T G C A G A C T A G T C G T A C T C A G C G T A A G C T A C G T G C T A G T A C | Pax7(Paired,Homeobox),long/Myoblast-Pax7-ChIP-Seq(GSE25064)/Homer | 1e-17 | -4.030e+01 | 0.0000 | 396.0 | 0.94% | 64.1 | 0.59% | motif file (matrix) | svg |
| 172 | T A C G T A G C G C T A C G A T C T A G A C G T C A G T C A G T G C T A A G T C G T C A G C A T | FOXK2(Forkhead)/U2OS-FOXK2-ChIP-Seq(E-MTAB-2204)/Homer | 1e-17 | -4.020e+01 | 0.0000 | 8792.0 | 20.84% | 2084.7 | 19.18% | motif file (matrix) | svg |
| 173 | G T C A C T G A T C A G G A T C T G C A T G C A A C G T T C A G C G T A C G T A C G T A G C T A | Hoxd12(Homeobox)/ChickenMSG-Hoxd12.Flag-ChIP-Seq(GSE86088)/Homer | 1e-17 | -4.006e+01 | 0.0000 | 22340.0 | 52.96% | 5529.3 | 50.87% | motif file (matrix) | svg |
| 174 | A C T G G A T C G A C T A C T G A C G T C A T G A C T G A C G T A G C T C G A T | RUNX-AML(Runt)/CD4+-PolII-ChIP-Seq(Barski\_et\_al.)/Homer | 1e-17 | -4.000e+01 | 0.0000 | 8064.0 | 19.12% | 1903.0 | 17.51% | motif file (matrix) | svg |
| 175 | A T C G A G C T A C T G A G T C A C T G A G T C C G T A A C G T A C T G A G T C A C T G A G T C | NRF(NRF)/Promoter/Homer | 1e-17 | -3.995e+01 | 0.0000 | 922.0 | 2.19% | 178.0 | 1.64% | motif file (matrix) | svg |
| 176 | C T G A T A C G G C A T A G C T A G C T A G T C T C G A A C T G C A G T A G C T A G C T G A T C | IRF3(IRF)/BMDM-Irf3-ChIP-Seq(GSE67343)/Homer | 1e-16 | -3.899e+01 | 0.0000 | 4730.0 | 11.21% | 1082.5 | 9.96% | motif file (matrix) | svg |
| 177 | C G T A C T A G T C A G T C A G A G T C A T G C A G T C G C A T A G C T A C G T A T C G C G A T | Sox9(HMG)/Limb-SOX9-ChIP-Seq(GSE73225)/Homer | 1e-16 | -3.895e+01 | 0.0000 | 9883.0 | 23.43% | 2360.7 | 21.72% | motif file (matrix) | svg |
| 178 | A C T G A G C T A G T C G T C A A G C T T C A G A T G C G A T C G C A T A T C G T C G A T A G C C G A T C A T G T A G C | Pax8(Paired,Homeobox)/Thyroid-Pax8-ChIP-Seq(GSE26938)/Homer | 1e-16 | -3.710e+01 | 0.0000 | 2847.0 | 6.75% | 629.3 | 5.79% | motif file (matrix) | svg |
| 179 | T C G A T G C A A C T G G T C A A G T C A G T C A G T C A G T C A G C T T G A C | LRF(Zf)/Erythroblasts-ZBTB7A-ChIP-Seq(GSE74977)/Homer | 1e-15 | -3.644e+01 | 0.0000 | 7589.0 | 17.99% | 1793.9 | 16.50% | motif file (matrix) | svg |
| 180 | C T G A C G A T C T A G T C A G G A T C C T G A T C A G G A T C C T G A A C T G A G T C G C T A A C G T A G T C G C A T | PRDM9(Zf)/Testis-DMC1-ChIP-Seq(GSE35498)/Homer | 1e-15 | -3.513e+01 | 0.0000 | 5043.0 | 11.95% | 1166.9 | 10.74% | motif file (matrix) | svg |
| 181 | G C T A C G A T C T A G G T A C G C T A C G A T C G T A G C T A C G A T C A G T G A T C G C T A | Pit1+1bp(Homeobox)/GCrat-Pit1-ChIP-Seq(GSE58009)/Homer | 1e-14 | -3.317e+01 | 0.0000 | 6036.0 | 14.31% | 1415.8 | 13.02% | motif file (matrix) | svg |
| 182 | A G C T A G T C A G T C A C G T C T A G A C G T A C G T A C G T C G T A A G T C G A T C C G T A | FOXP1(Forkhead)/H9-FOXP1-ChIP-Seq(GSE31006)/Homer | 1e-14 | -3.301e+01 | 0.0000 | 6422.0 | 15.22% | 1511.7 | 13.91% | motif file (matrix) | svg |
| 183 | T C A G T C A G G C T A C G T A T A C G G A C T T C A G T C G A C T G A C G T A T A C G G A C T | IRF8(IRF)/BMDM-IRF8-ChIP-Seq(GSE77884)/Homer | 1e-14 | -3.301e+01 | 0.0000 | 4157.0 | 9.85% | 953.3 | 8.77% | motif file (matrix) | svg |
| 184 | G C T A A T C G G C T A G A C T G C T A T C G A T A G C T C G A | GATA3(Zf)/iTreg-Gata3-ChIP-Seq(GSE20898)/Homer | 1e-14 | -3.282e+01 | 0.0000 | 17231.0 | 40.85% | 4240.9 | 39.01% | motif file (matrix) | svg |
| 185 | G A C T G A C T T C G A C G T A C G A T A G C T C T G A A C T G T G A C G A C T T C G A C G T A A C G T A G C T C T G A C T G A G T C A G C T A G C T A C G T A | Pax7(Paired,Homeobox),longest/Myoblast-Pax7-ChIP-Seq(GSE25064)/Homer | 1e-13 | -3.204e+01 | 0.0000 | 468.0 | 1.11% | 83.2 | 0.77% | motif file (matrix) | svg |
| 186 | A G T C C G A T A C T G A T C G T G A C G C T A C A T G A T C G T G A C C G A T A C T G T A G C G T A C G T C A | Tlx?(NR)/NPC-H3K4me1-ChIP-Seq(GSE16256)/Homer | 1e-13 | -3.176e+01 | 0.0000 | 4816.0 | 11.42% | 1117.0 | 10.28% | motif file (matrix) | svg |
| 187 | A G T C A T C G G C A T C T A G A C T G T A C G C G A T T C A G C A T G A G C T T A G C G A T C | GLI3(Zf)/Limb-GLI3-ChIP-Chip(GSE11077)/Homer | 1e-13 | -3.126e+01 | 0.0000 | 874.0 | 2.07% | 173.4 | 1.60% | motif file (matrix) | svg |
| 188 | C A T G A G T C C T G A T G A C A T C G G A C T G T C A A G T C T A G C G A T C | HIF2a(bHLH)/785\_O-HIF2a-ChIP-Seq(GSE34871)/Homer | 1e-13 | -3.113e+01 | 0.0000 | 2739.0 | 6.49% | 612.4 | 5.63% | motif file (matrix) | svg |
| 189 | C A T G T G A C C G T A A G T C T A C G G C A T A C T G G T C A A T G C A G T C | bHLHE41(bHLH)/proB-Bhlhe41-ChIP-Seq(GSE93764)/Homer | 1e-13 | -3.097e+01 | 0.0000 | 8994.0 | 21.32% | 2158.9 | 19.86% | motif file (matrix) | svg |
| 190 | C G T A A C T G C G T A A C G T C G T A C T G A T C A G T G C A A G C T T G A C | TRPS1(Zf)/MCF7-TRPS1-ChIP-Seq(GSE107013)/Homer | 1e-13 | -3.032e+01 | 0.0000 | 22403.0 | 53.11% | 5577.6 | 51.31% | motif file (matrix) | svg |
| 191 | C G T A T A C G T C G A A C T G A C T G C G T A C G T A T A C G A G C T T A C G | PU.1(ETS)/ThioMac-PU.1-ChIP-Seq(GSE21512)/Homer | 1e-13 | -3.032e+01 | 0.0000 | 5509.0 | 13.06% | 1291.5 | 11.88% | motif file (matrix) | svg |
| 192 | C T A G A C G T G C A T T C G A G T C A G C A T A T C G C G T A C A G T A G C T C T G A T G C A | HNF1b(Homeobox)/PDAC-HNF1B-ChIP-Seq(GSE64557)/Homer | 1e-12 | -2.971e+01 | 0.0000 | 2008.0 | 4.76% | 439.9 | 4.05% | motif file (matrix) | svg |
| 193 | T A C G A T G C G C T A A C T G C G T A A C G T C G T A C T G A T A C G T C G A | Gata4(Zf)/Heart-Gata4-ChIP-Seq(GSE35151)/Homer | 1e-12 | -2.917e+01 | 0.0000 | 11743.0 | 27.84% | 2856.2 | 26.28% | motif file (matrix) | svg |
| 194 | T A C G T C G A T A G C A G T C C G T A A G T C C T A G G C A T A C T G A T C G | n-Myc(bHLH)/mES-nMyc-ChIP-Seq(GSE11431)/Homer | 1e-12 | -2.886e+01 | 0.0000 | 6334.0 | 15.02% | 1499.9 | 13.80% | motif file (matrix) | svg |
| 195 | T C G A C T G A C T G A A T G C G T A C G T C A A G C T A G C T C G T A T C A G | Barx1(Homeobox)/Stomach-Barx1.3xFlag-ChIP-Seq(GSE69483)/Homer | 1e-12 | -2.837e+01 | 0.0000 | 7248.0 | 17.18% | 1728.8 | 15.90% | motif file (matrix) | svg |
| 196 | C T G A G T A C G A C T A G T C C A G T T G C A C T G A A C G T A G C T G A T C C T A G C G A T A C T G A T G C G A C T C T G A G A T C G A C T A G C T G A T C | Mouse\_Recombination\_Hotspot(Zf)/Testis-DMC1-ChIP-Seq(GSE24438)/Homer | 1e-12 | -2.823e+01 | 0.0000 | 889.0 | 2.11% | 179.3 | 1.65% | motif file (matrix) | svg |
| 197 | A C T G A C G T C A T G A T C G A T C G T G A C A C T G A T C G A T C G T G C A C T G A C G T A | E2F3(E2F)/MEF-E2F3-ChIP-Seq(GSE71376)/Homer | 1e-12 | -2.794e+01 | 0.0000 | 2850.0 | 6.76% | 644.4 | 5.93% | motif file (matrix) | svg |
| 198 | A C G T C T G A G A T C A T C G G A C T T C A G G T A C T A G C | HIF-1a(bHLH)/MCF7-HIF1a-ChIP-Seq(GSE28352)/Homer | 1e-11 | -2.659e+01 | 0.0000 | 1801.0 | 4.27% | 394.1 | 3.63% | motif file (matrix) | svg |
| 199 | A G T C C T G A A T C G A G C T A G C T G A C T A G T C G C T A A C G T C G A T G C A T C G A T A T C G C G T A T A G C G C A T A T G C C G T A | bZIP:IRF(bZIP,IRF)/Th17-BatF-ChIP-Seq(GSE39756)/Homer | 1e-11 | -2.588e+01 | 0.0000 | 6837.0 | 16.21% | 1632.9 | 15.02% | motif file (matrix) | svg |
| 200 | C T A G T A C G G A C T T G C A T G C A C G A T T A C G C T G A T C G A C T G A | Hoxa10(Homeobox)/ChickenMSG-Hoxa10.Flag-ChIP-Seq(GSE86088)/Homer | 1e-11 | -2.584e+01 | 0.0000 | 8139.0 | 19.29% | 1958.0 | 18.01% | motif file (matrix) | svg |
| 201 | C A T G A C G T A G T C G A T C G A T C G A T C G C T A C T A G C T A G C T A G T C A G T C G A | EBF1(EBF)/Near-E2A-ChIP-Seq(GSE21512)/Homer | 1e-11 | -2.576e+01 | 0.0000 | 7803.0 | 18.50% | 1874.9 | 17.25% | motif file (matrix) | svg |
| 202 | C T A G C A T G T G C A A C G T A G T C C G T A C A T G T C A G A C G T A C G T G C T A A G T C | Six1(Homeobox)/Myoblast-Six1-ChIP-Chip(GSE20150)/Homer | 1e-11 | -2.570e+01 | 0.0000 | 2978.0 | 7.06% | 679.9 | 6.25% | motif file (matrix) | svg |
| 203 | T C A G C T A G C T A G C T A G T C A G T C G A C T G A C G A T A G T C G A T C A G T C T G A C | NFkB-p50,p52(RHD)/Monocyte-p50-ChIP-Chip(Schreiber\_et\_al.)/Homer | 1e-11 | -2.544e+01 | 0.0000 | 541.0 | 1.28% | 103.5 | 0.95% | motif file (matrix) | svg |
| 204 | T G A C G T C A A G T C G C T A C T A G A G T C C T G A C A T G A C T G C T A G T A C G T C A G | Zic2(Zf)/ESC-Zic2-ChIP-Seq(SRP197560)/Homer | 1e-10 | -2.419e+01 | 0.0000 | 3641.0 | 8.63% | 844.3 | 7.77% | motif file (matrix) | svg |
| 205 | C T A G T A C G G A T C G T C A T C G A A C G T T C A G C G T A C G T A C G T A | Hoxd10(Homeobox)/ChickenMSG-Hoxd10.Flag-ChIP-Seq(GSE86088)/Homer | 1e-10 | -2.356e+01 | 0.0000 | 14659.0 | 34.75% | 3616.3 | 33.27% | motif file (matrix) | svg |
| 206 | T A G C C G T A C T G A T A C G C G T A A C G T A C T G A C T G A G T C T A C G C T A G G T A C | YY1(Zf)/Promoter/Homer | 1e-9 | -2.193e+01 | 0.0000 | 389.0 | 0.92% | 72.1 | 0.66% | motif file (matrix) | svg |
| 207 | C G T A C T G A A C T G A G C T A G T C G A T C G A T C G C A T C T G A C T A G C T A G T A C G T C G A T G C A G C A T | EBF2(EBF)/BrownAdipose-EBF2-ChIP-Seq(GSE97114)/Homer | 1e-9 | -2.169e+01 | 0.0000 | 8013.0 | 19.00% | 1939.4 | 17.84% | motif file (matrix) | svg |
| 208 | C G T A T A G C A G T C C T A G C A G T C T A G C T G A G T A C G C A T T C G A C G T A G C A T A G C T C T A G T C G A | PAX3:FKHR-fusion(Paired,Homeobox)/Rh4-PAX3:FKHR-ChIP-Seq(GSE19063)/Homer | 1e-9 | -2.129e+01 | 0.0000 | 2972.0 | 7.05% | 686.7 | 6.32% | motif file (matrix) | svg |
| 209 | A G T C G A C T A G C T C G A T A T C G G C T A C G A T A T C G C G A T A C T G T A C G A C G T | Tcf7(HMG)/GM12878-TCF7-ChIP-Seq(Encode)/Homer | 1e-8 | -2.004e+01 | 0.0000 | 4219.0 | 10.00% | 996.7 | 9.17% | motif file (matrix) | svg |
| 210 | G A T C C G T A G A C T C T A G G A T C C T G A G A C T C T G A G A C T C T A G G A T C C T G A G A C T C T G A G A C T | OCT:OCT(POU,Homeobox)/NPC-OCT6-ChIP-Seq(GSE43916)/Homer | 1e-8 | -1.980e+01 | 0.0000 | 2366.0 | 5.61% | 542.0 | 4.99% | motif file (matrix) | svg |
| 211 | T G C A T G C A A G T C A G T C G A C T C A G T A T G C G A T C C T G A A C G T C T A G C T A G A G T C A C G T A G T C A G T C A G T C G A C T C G T A A C G T A G C T C T A G G A T C G A T C G A T C | ZNF16(Zf)/HEK293-ZNF16.GFP-ChIP-Seq(GSE58341)/Homer | 1e-8 | -1.877e+01 | 0.0000 | 62.0 | 0.15% | 7.5 | 0.07% | motif file (matrix) | svg |
| 212 | C G A T G A C T C G A T T C A G G A C T A C G T C A G T C T G A G A C T G A C T A G C T C G A T A C T G A T C G G T A C G C T A | NF1:FOXA1(CTF,Forkhead)/LNCAP-FOXA1-ChIP-Seq(GSE27824)/Homer | 1e-7 | -1.826e+01 | 0.0000 | 803.0 | 1.90% | 169.5 | 1.56% | motif file (matrix) | svg |
| 213 | C A G T G A C T C G T A G C T A G T A C G A T C G T A C G A C T A G C T A C G T T G A C C G T A C A G T A C G T A T G C | ZNF652/HepG2-ZNF652.Flag-ChIP-Seq(Encode)/Homer | 1e-7 | -1.791e+01 | 0.0000 | 2898.0 | 6.87% | 675.8 | 6.22% | motif file (matrix) | svg |
| 214 | C G T A A C T G C G T A A C G T A T C G C A G T T A G C C G T A T C G A G T A C C T G A T A G C C G T A A C T G C G T A A C G T C G T A C T G A A T C G G C T A | GATA3(Zf),DR8/iTreg-Gata3-ChIP-Seq(GSE20898)/Homer | 1e-7 | -1.730e+01 | 0.0000 | 1066.0 | 2.53% | 232.5 | 2.14% | motif file (matrix) | svg |
| 215 | C T G A C G A T C T A G C G T A A G C T C G A T C A G T C T G A G A C T C T A G C T A G A T G C | PBX2(Homeobox)/K562-PBX2-ChIP-Seq(Encode)/Homer | 1e-7 | -1.699e+01 | 0.0000 | 11240.0 | 26.65% | 2772.6 | 25.51% | motif file (matrix) | svg |
| 216 | C T A G A T C G G A C T A G T C A G T C A G T C G A C T C T G A A C T G C T A G A C T G C T G A | EBF(EBF)/proBcell-EBF-ChIP-Seq(GSE21978)/Homer | 1e-7 | -1.638e+01 | 0.0000 | 1702.0 | 4.03% | 386.3 | 3.55% | motif file (matrix) | svg |
| 217 | G A C T C T A G C T A G C T A G A C T G T C G A C T G A C T A G C T A G C T A G G T A C G T C A | ZNF467(Zf)/HEK293-ZNF467.GFP-ChIP-Seq(GSE58341)/Homer | 1e-6 | -1.580e+01 | 0.0000 | 6714.0 | 15.92% | 1632.1 | 15.01% | motif file (matrix) | svg |
| 218 | G A C T G T A C T G C A A C G T G A T C G C T A T C G A A C G T A G T C C G T A | Pdx1(Homeobox)/Islet-Pdx1-ChIP-Seq(SRA008281)/Homer | 1e-6 | -1.577e+01 | 0.0000 | 12968.0 | 30.74% | 3217.3 | 29.60% | motif file (matrix) | svg |
| 219 | T C G A A C T G A C T G C G T A C G T A T C G A A G T C C T G A A T C G G T A C G C A T C A T G | ETS:E-box(ETS,bHLH)/HPC7-Scl-ChIP-Seq(GSE22178)/Homer | 1e-6 | -1.569e+01 | 0.0000 | 1511.0 | 3.58% | 341.4 | 3.14% | motif file (matrix) | svg |
| 220 | A G C T T A C G C G A T T C A G A G C T A C G T G A T C C T G A T A G C A G C T A T G C C T G A C G T A A T C G A G T C C T A G A C T G C T G A G T C A T C G A | PAX6(Paired,Homeobox)/Forebrain-Pax6-ChIP-Seq(GSE66961)/Homer | 1e-6 | -1.560e+01 | 0.0000 | 1052.0 | 2.49% | 231.3 | 2.13% | motif file (matrix) | svg |
| 221 | A C T G G A T C C T G A A T C G A G T C T A G C C T G A C G T A T A C G A G T C C T A G C A G T T C A G T C G A T G A C G A T C | PAX5(Paired,Homeobox)/GM12878-PAX5-ChIP-Seq(GSE32465)/Homer | 1e-6 | -1.551e+01 | 0.0000 | 3151.0 | 7.47% | 743.7 | 6.84% | motif file (matrix) | svg |
| 222 | A T C G T G A C A T G C C T G A T C A G G A C T A G T C C G A T T C A G T C G A C A T G C T A G C T A G C G T A C T A G C T A G C T G A C T A G C T A G A T G C | ZSCAN22(Zf)/HEK293-ZSCAN22.GFP-ChIP-Seq(GSE58341)/Homer | 1e-6 | -1.533e+01 | 0.0000 | 626.0 | 1.48% | 131.8 | 1.21% | motif file (matrix) | svg |
| 223 | C G A T C T G A C G T A A G C T A G T C T G C A C T G A A C G T A G C T G C T A | Pax7(Paired,Homeobox)/Myoblast-Pax7-ChIP-Seq(GSE25064)/Homer | 1e-6 | -1.532e+01 | 0.0000 | 1438.0 | 3.41% | 324.7 | 2.99% | motif file (matrix) | svg |
| 224 | G A C T C G A T C T G A G T C A G A C T C G A T T C G A C G T A G C T A G C T A T G A C G T A C C G T A A C T G T G C A C G A T A C T G A C G T | Pitx1:Ebox(Homeobox,bHLH)/Hindlimb-Pitx1-ChIP-Seq(GSE41591)/Homer | 1e-6 | -1.469e+01 | 0.0000 | 4201.0 | 9.96% | 1006.3 | 9.26% | motif file (matrix) | svg |
| 225 | T G A C G C T A T G A C C G T A T C A G G A T C C G T A C A T G C A T G C T A G C T A G C T A G | Unknown-ESC-element(?)/mES-Nanog-ChIP-Seq(GSE11724)/Homer | 1e-6 | -1.452e+01 | 0.0000 | 5781.0 | 13.70% | 1402.7 | 12.90% | motif file (matrix) | svg |
| 226 | C G T A T A G C T A G C T G C A A C T G C T A G C G T A C G T A T C A G G A C T | EHF(ETS)/LoVo-EHF-ChIP-Seq(GSE49402)/Homer | 1e-6 | -1.430e+01 | 0.0000 | 14504.0 | 34.38% | 3617.0 | 33.27% | motif file (matrix) | svg |
| 227 | A T C G A G T C A G T C G A C T A T G C C T G A C T A G A C T G T A C G G T A C C T G A C G A T | AP-2gamma(AP2)/MCF7-TFAP2C-ChIP-Seq(GSE21234)/Homer | 1e-5 | -1.378e+01 | 0.0000 | 6065.0 | 14.38% | 1476.1 | 13.58% | motif file (matrix) | svg |
| 228 | A T G C A G C T T C A G T G A C T C A G A T G C T G C A A C G T A T C G G A T C A C T G A G T C | NRF1(NRF)/MCF7-NRF1-ChIP-Seq(Unpublished)/Homer | 1e-5 | -1.338e+01 | 0.0000 | 383.0 | 0.91% | 77.5 | 0.71% | motif file (matrix) | svg |
| 229 | A C T G C A T G G C T A T C G A G C T A A G C T A G C T G T A C A G T C T G A C | NFkB-p65-Rel(RHD)/ThioMac-LPS-Expression(GSE23622)/Homer | 1e-5 | -1.246e+01 | 0.0000 | 578.0 | 1.37% | 123.2 | 1.13% | motif file (matrix) | svg |
| 230 | C T G A A T G C C G T A A C G T A G T C A G T C A C G T A C T G A T C G G C A T | SPDEF(ETS)/VCaP-SPDEF-ChIP-Seq(SRA014231)/Homer | 1e-5 | -1.184e+01 | 0.0000 | 11026.0 | 26.14% | 2741.6 | 25.22% | motif file (matrix) | svg |
| 231 | C T A G T C G A C G A T C T A G G C A T C A G T C T A G G A T C C G T A G T C A | CEBP:AP1(bZIP)/ThioMac-CEBPb-ChIP-Seq(GSE21512)/Homer | 1e-5 | -1.176e+01 | 0.0000 | 10156.0 | 24.08% | 2520.4 | 23.19% | motif file (matrix) | svg |
| 232 | A C G T T G C A A G C T G A T C C T A G C T G A A G C T G T C A T C G A C G T A | CUX1(Homeobox)/K562-CUX1-ChIP-Seq(GSE92882)/Homer | 1e-5 | -1.165e+01 | 0.0000 | 7861.0 | 18.64% | 1938.7 | 17.83% | motif file (matrix) | svg |
| 233 | G T A C G C T A T C A G C T G A C T A G C A T G A G C T G A T C T G C A T C G A C T G A A C T G C A G T A G T C G A T C G C T A | HNF4a(NR),DR1/HepG2-HNF4a-ChIP-Seq(GSE25021)/Homer | 1e-4 | -1.145e+01 | 0.0000 | 4036.0 | 9.57% | 975.0 | 8.97% | motif file (matrix) | svg |
| 234 | T G A C C G A T C T G A C T A G C T A G A C G T A T G C T G C A T C G A C T G A C T A G C A T G A C G T A G T C C G T A | PPARa(NR),DR1/Liver-Ppara-ChIP-Seq(GSE47954)/Homer | 1e-4 | -1.121e+01 | 0.0000 | 8563.0 | 20.30% | 2118.1 | 19.49% | motif file (matrix) | svg |
| 235 | A T G C A G T C A G C T A G C T A C G T A T C G C G T A C G A T T A G C G A C T | LEF1(HMG)/H1-LEF1-ChIP-Seq(GSE64758)/Homer | 1e-4 | -1.115e+01 | 0.0000 | 8885.0 | 21.06% | 2200.2 | 20.24% | motif file (matrix) | svg |
| 236 | A T C G T C G A G A C T A T C G T G A C A C G T C T A G A C T G C G T A A C T G A G T C G T A C | ZNF415(Zf)/HEK293-ZNF415.GFP-ChIP-Seq(GSE58341)/Homer | 1e-4 | -1.110e+01 | 0.0000 | 4494.0 | 10.65% | 1091.4 | 10.04% | motif file (matrix) | svg |
| 237 | T A C G C T A G A T G C G A T C G T A C A G T C C T A G A G T C A G T C A G T C G T A C A G T C | Sp1(Zf)/Promoter/Homer | 1e-4 | -1.103e+01 | 0.0000 | 630.0 | 1.49% | 138.0 | 1.27% | motif file (matrix) | svg |
| 238 | C A T G G A C T G C A T A C T G A G C T A C T G A C T G C G T A G C A T A G C T A T C G T A C G | Foxh1(Forkhead)/hESC-FOXH1-ChIP-Seq(GSE29422)/Homer | 1e-4 | -1.103e+01 | 0.0000 | 7842.0 | 18.59% | 1936.1 | 17.81% | motif file (matrix) | svg |
| 239 | A T G C G A C T A G C T A G C T A G T C G C T A C A G T C G A T G C T A A C G T A C T G G C T A T A G C G C A T T G A C | IRF:BATF(IRF:bZIP)/pDC-Irf8-ChIP-Seq(GSE66899)/Homer | 1e-4 | -1.090e+01 | 0.0000 | 1845.0 | 4.37% | 432.8 | 3.98% | motif file (matrix) | svg |
| 240 | C T A G C T A G C G T A C G T A T A C G C G A T C T A G C T G A C T G A C G T A T A C G G A C T | PU.1:IRF8(ETS:IRF)/pDC-Irf8-ChIP-Seq(GSE66899)/Homer | 1e-4 | -1.053e+01 | 0.0000 | 2428.0 | 5.76% | 577.9 | 5.32% | motif file (matrix) | svg |
| 241 | T A C G T C A G T G C A A G C T T G A C A G C T A G T C A C T G G A T C A C T G T C G A A C T G C T G A C T G A A T G C | ZBTB33(Zf)/GM12878-ZBTB33-ChIP-Seq(GSE32465)/Homer | 1e-4 | -1.046e+01 | 0.0001 | 51.0 | 0.12% | 7.5 | 0.07% | motif file (matrix) | svg |
| 242 | C T A G A C G T A G T C C G T A A C T G A G T C G C A T A C T G G C A T A G T C G A C T G A T C G C A T A G T C A G C T | ZNF317(Zf)/HEK293-ZNF317.GFP-ChIP-Seq(GSE58341)/Homer | 1e-4 | -1.038e+01 | 0.0001 | 1051.0 | 2.49% | 239.4 | 2.20% | motif file (matrix) | svg |
| 243 | C T G A T C A G A C T G C A G T A G T C G A C T A G T C A C G T C G T A G C T A A T G C G A T C | PRDM14(Zf)/H1-PRDM14-ChIP-Seq(GSE22767)/Homer | 1e-4 | -1.008e+01 | 0.0001 | 3178.0 | 7.53% | 765.7 | 7.04% | motif file (matrix) | svg |
| 244 | G A C T A G C T G T A C G A C T C T G A A C T G G T C A C T G A A T G C T A C G G A C T A C G T A G T C G A C T C T G A | HRE(HSF)/Striatum-HSF1-ChIP-Seq(GSE38000)/Homer | 1e-4 | -1.004e+01 | 0.0001 | 2937.0 | 6.96% | 705.6 | 6.49% | motif file (matrix) | svg |
| 245 | C T G A A G T C C G A T A G C T A T G C G T A C A C G T A T C G C A G T G C A T | Elf4(ETS)/BMDM-Elf4-ChIP-Seq(GSE88699)/Homer | 1e-4 | -9.919e+00 | 0.0001 | 10586.0 | 25.09% | 2639.8 | 24.29% | motif file (matrix) | svg |
| 246 | T G A C C T G A C T A G C T G A C G T A A G T C C T G A A C G T G C A T T A G C G C A T A T C G G A C T G A C T G A T C | GRE(NR),IR3/RAW264.7-GRE-ChIP-Seq(Unpublished)/Homer | 1e-4 | -9.850e+00 | 0.0001 | 3191.0 | 7.56% | 769.2 | 7.08% | motif file (matrix) | svg |
| 247 | T C G A T A G C G T C A A C T G A C T G C G T A C G T A C T A G A G C T T C A G | ERG(ETS)/VCaP-ERG-ChIP-Seq(GSE14097)/Homer | 1e-4 | -9.456e+00 | 0.0001 | 16577.0 | 39.30% | 4174.8 | 38.41% | motif file (matrix) | svg |
| 248 | T A C G C T G A T C G A C G A T C T A G C T A G T C G A C T G A T C G A T C G A C G T A T C G A G C A T C A T G C G T A T A C G G C A T T G A C C G T A A G C T | NFAT:AP1(RHD,bZIP)/Jurkat-NFATC1-ChIP-Seq(Jolma\_et\_al.)/Homer | 1e-4 | -9.367e+00 | 0.0002 | 2471.0 | 5.86% | 591.6 | 5.44% | motif file (matrix) | svg |
| 249 | C A G T A C T G C G T A G T C A C G T A A G C T G A T C A G C T A C T G C G T A A G T C G T C A A G T C A G T C A T C G | Six4(Homeobox)/MCF7-SIX4-ChIP-Seq(Encode)/Homer | 1e-4 | -9.351e+00 | 0.0002 | 532.0 | 1.26% | 116.8 | 1.07% | motif file (matrix) | svg |
| 250 | G T A C C T A G T C A G A G C T T A G C C G T A A T G C T A C G A G T C G T A C G T C A A G T C | Srebp2(bHLH)/HepG2-Srebp2-ChIP-Seq(GSE31477)/Homer | 1e-3 | -9.121e+00 | 0.0002 | 1029.0 | 2.44% | 236.7 | 2.18% | motif file (matrix) | svg |
| 251 | T C A G C G T A A G T C A G C T C G T A A G T C C T G A C G T A A G T C G C A T A G T C A G T C A G T C C T G A A C T G T G C A T C G A C A T G A T C G G A T C | Ronin(THAP)/ES-Thap11-ChIP-Seq(GSE51522)/Homer | 1e-3 | -8.635e+00 | 0.0003 | 83.0 | 0.20% | 14.4 | 0.13% | motif file (matrix) | svg |
| 252 | C G T A A C T G G T C A A C G T A T C G C A G T C T A G T C A G C G T A A C T G C G T A A C G T C G T A C T G A T A C G | GATA3(Zf),DR4/iTreg-Gata3-ChIP-Seq(GSE20898)/Homer | 1e-3 | -8.291e+00 | 0.0004 | 1198.0 | 2.84% | 279.5 | 2.57% | motif file (matrix) | svg |
| 253 | A G C T C T G A C T A G C T A G A C T G T A G C T G C A T C G A C T G A C T A G C A T G A C G T A T G C T C G A | RXR(NR),DR1/3T3L1-RXR-ChIP-Seq(GSE13511)/Homer | 1e-3 | -8.165e+00 | 0.0005 | 8525.0 | 20.21% | 2124.7 | 19.55% | motif file (matrix) | svg |
| 254 | T C A G A T C G G A C T A C T G G A C T C A G T C T A G C G T A G T A C C G T A C T A G A T C G | Tbx20(T-box)/Heart-Tbx20-ChIP-Seq(GSE29636)/Homer | 1e-3 | -7.987e+00 | 0.0006 | 2678.0 | 6.35% | 647.6 | 5.96% | motif file (matrix) | svg |
| 255 | A G T C A T G C C G A T A C T G C G T A A C G T A G C T A G T C C G T A C G T A A C G T A G T C G C T A G C T A T G C A | DUX(Homeobox)/C2C12-Dux-ChIP-Seq(GSE87279)/Homer | 1e-3 | -7.977e+00 | 0.0006 | 47.0 | 0.11% | 7.6 | 0.07% | motif file (matrix) | svg |
| 256 | G A C T A G T C C G A T A C T G C T G A T G A C G T A C C G T A A T C G G C A T C T G A C T A G | Bcl11a(Zf)/HSPC-BCL11A-ChIP-Seq(GSE104676)/Homer | 1e-3 | -7.964e+00 | 0.0006 | 5921.0 | 14.04% | 1464.1 | 13.47% | motif file (matrix) | svg |
| 257 | C T G A C T A G T C G A C G T A A T G C C G T A A T C G C G A T T A G C G C A T A T C G G C A T A G C T G A T C G A C T A G C T | ARE(NR)/LNCAP-AR-ChIP-Seq(GSE27824)/Homer | 1e-3 | -7.896e+00 | 0.0006 | 3132.0 | 7.42% | 761.6 | 7.01% | motif file (matrix) | svg |
| 258 | T A C G C G T A T C A G G A C T C T A G A C T G C A G T T A G C T C G A A C G T G T A C C T A G A G T C A G T C G A T C | ZNF669(Zf)/HEK293-ZNF669.GFP-ChIP-Seq(GSE58341)/Homer | 1e-3 | -7.315e+00 | 0.0011 | 453.0 | 1.07% | 100.1 | 0.92% | motif file (matrix) | svg |
| 259 | T A G C G A T C A G C T T G A C G C T A A G C T C A T G A C T G A C G T T C A G A G T C G A T C G A C T A G C T G C T A A G T C A G C T A G T C G A T C A T G C A G C T G A C T C A T G A C G T A T C G | ZNF41(Zf)/HEK293-ZNF41.GFP-ChIP-Seq(GSE58341)/Homer | 1e-3 | -7.293e+00 | 0.0012 | 275.0 | 0.65% | 58.8 | 0.54% | motif file (matrix) | svg |
| 260 | T A C G C T G A C A T G G A T C G T A C G C A T T C A G T A C G A G C T G T C A G A T C G C A T T A C G C G T A C T A G G A T C G A T C C G A T A C T G T C A G | ZNF322(Zf)/HEK293-ZNF322.GFP-ChIP-Seq(GSE58341)/Homer | 1e-3 | -7.184e+00 | 0.0013 | 2243.0 | 5.32% | 541.6 | 4.98% | motif file (matrix) | svg |
| 261 | C T A G T A C G G A T C G T C A T G C A A C G T T G C A G C T A T C G A T G C A | Hoxa9(Homeobox)/ChickenMSG-Hoxa9.Flag-ChIP-Seq(GSE86088)/Homer | 1e-3 | -7.025e+00 | 0.0015 | 27971.0 | 66.31% | 7129.6 | 65.59% | motif file (matrix) | svg |
| 262 | A C T G C T A G G A T C C G T A C T A G A G T C T G C A G A C T C G T A A C G T T C A G A G T C A C G T C T G A A G T C A G T C G A T C C G T A T C A G T A C G | EBNA1(EBV-virus)/Raji-EBNA1-ChIP-Seq(GSE30709)/Homer | 1e-3 | -6.995e+00 | 0.0015 | 143.0 | 0.34% | 28.9 | 0.27% | motif file (matrix) | svg |
| 263 | T C G A G A C T A G C T T G A C A G C T G T A C T C A G G A T C A T C G T G C A A C T G C T G A | GFX(?)/Promoter/Homer | 1e-2 | -6.768e+00 | 0.0019 | 18.0 | 0.04% | 2.3 | 0.02% | motif file (matrix) | svg |
| 264 | T A C G G A C T A C T G A C T G C T A G A T G C A G T C A G T C A G T C C T G A | ZNF692(Zf)/HEK293-ZNF692.GFP-ChIP-Seq(GSE58341)/Homer | 1e-2 | -6.763e+00 | 0.0019 | 694.0 | 1.65% | 159.5 | 1.47% | motif file (matrix) | svg |
| 265 | T G C A C G T A A C T G T C A G C A G T C A T G T C A G G A T C T A C G A G T C T G C A A C T G A C T G T G A C G T C A | ZNF165(Zf)/WHIM12-ZNF165-ChIP-Seq(GSE65937)/Homer | 1e-2 | -6.638e+00 | 0.0022 | 771.0 | 1.83% | 178.7 | 1.64% | motif file (matrix) | svg |
| 266 | T G C A A C T G T A C G A T G C A G T C G A C T T C G A A T C G | ZNF711(Zf)/SHSY5Y-ZNF711-ChIP-Seq(GSE20673)/Homer | 1e-2 | -6.406e+00 | 0.0027 | 10329.0 | 24.49% | 2595.8 | 23.88% | motif file (matrix) | svg |
| 267 | G C T A T A G C A G C T A T C G G T C A C G T A G C T A A T G C G A T C C T G A | IRF4(IRF)/GM12878-IRF4-ChIP-Seq(GSE32465)/Homer | 1e-2 | -6.070e+00 | 0.0038 | 5314.0 | 12.60% | 1320.2 | 12.15% | motif file (matrix) | svg |
| 268 | G C A T A T C G C A T G G T A C G C T A A G T C T C A G T G A C G T C A T G C A | Arnt:Ahr(bHLH)/MCF7-Arnt-ChIP-Seq(Lo\_et\_al.)/Homer | 1e-2 | -5.891e+00 | 0.0045 | 5708.0 | 13.53% | 1421.7 | 13.08% | motif file (matrix) | svg |
| 269 | C G T A C T G A C T A G C T G A A G T C G C T A C G A T A T C G G A C T G A T C A G T C C T G A C T A G C T A G A G T C G C T A C G A T C T A G G A T C G A T C | p73(p53)/Trachea-p73-ChIP-Seq(PRJNA310161)/Homer | 1e-2 | -5.418e+00 | 0.0073 | 416.0 | 0.99% | 94.9 | 0.87% | motif file (matrix) | svg |
| 270 | C T A G C A T G C A T G T A C G A G T C G C A T A G C T C T A G A C G T A G T C G A C T A C T G A C T G A C T G T C G A | Zfp809(Zf)/ES-Zfp809-ChIP-Seq(GSE70799)/Homer | 1e-2 | -5.070e+00 | 0.0102 | 1145.0 | 2.71% | 274.6 | 2.53% | motif file (matrix) | svg |
| 271 | G C A T C G T A C G A T G A C T A C T G C T G A G A C T G A T C | Hnf6b(Homeobox)/LNCaP-Hnf6b-ChIP-Seq(GSE106305)/Homer | 1e-2 | -5.032e+00 | 0.0106 | 9937.0 | 23.56% | 2505.3 | 23.05% | motif file (matrix) | svg |
| 272 | A T G C T A G C A G C T A G C T T G A C G A C T T C A G T A C G G T C A C T G A A T C G T A G C G A C T C A G T A G T C A G C T T C G A A T C G T G C A T G C A | HRE(HSF)/HepG2-HSF1-ChIP-Seq(GSE31477)/Homer | 1e-2 | -4.923e+00 | 0.0118 | 1676.0 | 3.97% | 407.3 | 3.75% | motif file (matrix) | svg |
| 273 | C G T A C T G A C G T A C T A G T C G A C T A G A C T G C G T A C G T A T A C G A G C T A T C G | SpiB(ETS)/OCILY3-SPIB-ChIP-Seq(GSE56857)/Homer | 1e-2 | -4.806e+00 | 0.0132 | 2708.0 | 6.42% | 667.4 | 6.14% | motif file (matrix) | svg |
| 274 | T A C G T A C G G T A C A T C G A C T G T A C G T C G A C T G A T C G A A T C G | E2F6(E2F)/Hela-E2F6-ChIP-Seq(GSE31477)/Homer | 1e-2 | -4.736e+00 | 0.0141 | 2318.0 | 5.49% | 569.1 | 5.24% | motif file (matrix) | svg |
| 275 | C T G A C T A G A C T G G C A T A T G C C G T A C T G A C T A G A C T G A C G T A G T C C T G A | RARg(NR)/ES-RARg-ChIP-Seq(GSE30538)/Homer | 1e-2 | -4.653e+00 | 0.0153 | 464.0 | 1.10% | 107.8 | 0.99% | motif file (matrix) | svg |
| 276 | T C G A C T A G A G T C A G T C C G T A C G T A A C G T T A G C T C A G T A C G | NFY(CCAAT)/Promoter/Homer | 1e-2 | -4.637e+00 | 0.0154 | 7549.0 | 17.90% | 1898.5 | 17.47% | motif file (matrix) | svg |
| 277 | T A G C T C A G C A T G G C A T A G C T C G A T A T G C C G T A C G T A G T C A | CHR(?)/Hela-CellCycle-Expression/Homer | 1e-2 | -4.623e+00 | 0.0156 | 9666.0 | 22.91% | 2439.9 | 22.45% | motif file (matrix) | svg |
